# OEPR Cloning: an Efficient and Seamless Cloning Strategy for Large- and Multi-Fragments

**DOI:** 10.1038/srep44648

**Published:** 2017-03-16

**Authors:** Chang-Jun Liu, Hui Jiang, Lei Wu, Ling-Yun Zhu, Er Meng, Dong-Yi Zhang

**Affiliations:** 1 Research Center of Biological Information, College of Science, National University of Defense Technology, Changsha, Hunan 410073, China; 2 Beijing Institute of Pharmaceutical Chemistry, Beijing 102205, China

## Abstract

Here, an efficient cloning strategy for large DNA fragments and for simultaneous assembly of multiple DNA fragments assembly is presented. This strategy is named OEPR (based on **O**verlap **E**xtension **P**CR and **R**ecombination *in vivo*). OEPR cloning is a seamless, restriction- and ligation-independent method. The method takes advantage of both homologous recombination enzymes in *E. coli* and overlap PCR. Using OEPR cloning, a long fragment (1–6 kb) or multiple fragments (2–4 fragments) can be easily constructed and simultaneously assembled into a target vector.

A basic and essential procedure in biological research is the cloning of DNA fragments into plasmids. Traditional cloning methods rely on digestion of DNA by Type II restriction enzymes and the *in vitro* ligation of DNA fragments to vectors. However, because the restriction and ligation steps are inefficient and time-consuming, the success rates of traditional cloning methods are low. In addition, by traditional cloning methods using restriction enzymes, unwanted nucleotide sequences may be added to inserts, which might result in undesired changes to the structure and activity of the translated products^1^. Consequently, seamless cloning methods (e.g., enzyme-free cloning^2^, hetero-stagger cloning^3^ and polymerase incomplete primer extension (PIPE) cloning^4^,^5^) have been developed, which utilize a compatible set of tailed and non-tailed primers to generate DNA fragments and linear vectors with cohesive ends. These DNA fragments and linear vectors can then be paired together and repaired after direct transformation into *E. coli*. Seamless cloning methods are restriction- and ligation-independent as well as easy to implement; however, seamless methods still require careful design of numerous primers to ensure successful amplification of DNA fragments and linearized vectors. Uracil DNA-glycosylase cloning (UDG)^6^,^7^ and Ligation-Independent Cloning (LIC)^8^, which take advantage of the 3′-5′ exonuclease activities of uracil DNA–glycosylase and T4 DNA polymerase, respectively, are able to generate 12-base cohesive ends.

Based on the principle of QuickChange^TM^ site-directed mutagenesis^9^,^10^, restriction-free (RF) cloning^11^,^12^ is a straightforward, efficient and reliable method for inserting a single fragment into a vector; this method does not introduce any unwanted extra bases at DNA fragment/insertion site junctions. However, RF cloning suffers from low DNA product yields due to linear amplification and low efficiencies for inserts longer than 1 kb. Although Exponential Megapriming PCR (EMP) Cloning^13^ and Inverse Fusion PCR Cloning (IFPC)^14^ address the product yield issue by introducing reverse primers to ensure exponential amplification, the phosphorylation and ligation of the final, second-round PCR products add to the labor and cost required for these methods.

If homologous regions exist between the linear inserts and vectors, *in vitro* recombination can be performed using commercially available recombinant enzymes such as Gateway^®^ (Invitrogen)^15^ and In-Fusion^TM^ (Clontech)^16^. Homologous recombination can also occur in *E. coli in vivo* via three different mechanisms: RecA-dependent, RecA-independent and Red/ET-dependent; these mechanisms require >1 kb, >12 bp and 30–50 bp of homology, respectively^1^. The RecA-dependent mechanism requires homology regions that are too long to be ideal for cloning and sub-cloning. Red/ET-dependent recombination, which is based on the function of 5′-3′ exonuclease and single-stranded DNA annealing protein pairs, is an efficient approach. However, expression of the Redα/Redβ or the RecE/RecT protein pair is required for successful Red/ET-dependent recombination^17^,^18^,^19^. High efficiencies can also be achieved by integrating the λ prophage Red recombination system into the *E. coli* genome^20^. In contrast, RecA-independent recombination occurs in RecA strains at low efficiencies. Recently, *in vivo* RecA-independent recombination-like cloning was demonstrated in RecA- strains, such as *E. coli* strains DH10B and JM109^20^,^21^,^22^. Although *in vitro* and *in vivo* homologous recombination is convenient, such mechanisms require expensive enzymes or specific strains. Inspired by the aforementioned cloning methods, we developed a hybrid seamless method based on **O**verlap **E**xtension **P**CR and **R**ecombination *in vivo* and named it OEPR cloning. With OEPR cloning, the assembly of large DNA fragments (1–6 kb) and simultaneous assembly of multiple fragments (up to 4) can be easily performed in a single day. Compared to homologous recombination, RF cloning or other similar methods, OEPR cloning is much more efficient and cost-effective for the insertion of longer fragments or the assembly of multiple fragments.

## Results

### Overview of OEPR cloning

**Figure 1:**
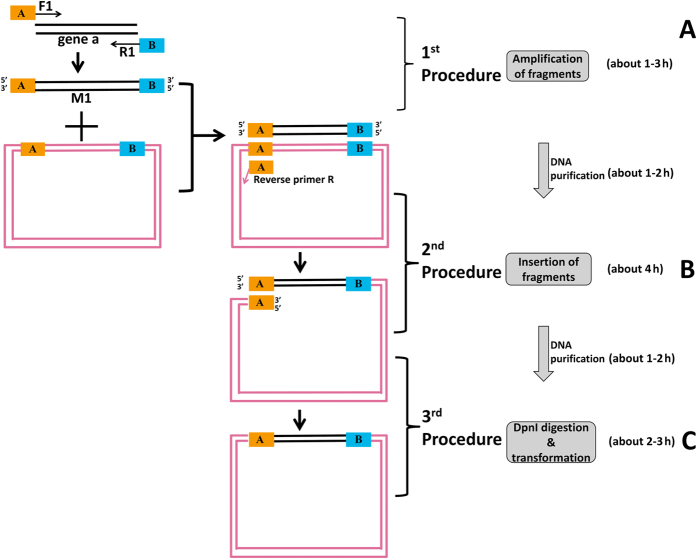
Schematic diagram and flowchart of OEPR cloning for large fragment insertion. The cloning method requires only three steps: (**A**) amplification of insert fragments using primer F1 and R1; (**B**) exponential amplification using fragments generated in the first PCR and reverse primer R; and (**C**) digestion by *DpnI* to eliminate the parent DNA and direct transformation into chemically competent Top10F’ cells.

The mechanism of OEPR cloning for insertion of single fragments is shown in Fig. 1. OEPR cloning requires three steps. (i) The first step of OEPR cloning is the exponential amplification of the target insert a using a primer pair with overhangs A and B (F1 and R1) (Fig. 1A). The resulting PCR product (M1) contains regions homologous to the insert part of the vector (overhangs A and B) (Fig. 1A). The 5′ end of the reverse primer R1 contains the homologous arm (sky blue part B, Fig. 1A) used for overlap extension PCR (Tm = 68–70 °C). The 5′ end of the forward primer (F1) contains the homologous region (orange part A, Fig. 1A) used *for in vivo* recombination. ii) In the second step, the PCR product M1 is used together with a short reverse primer (R) to exponentially amplify the target plasmid (Fig. 1B). As shown in Fig. 1B, a final PCR product containing homologous region A (in orange) is created after two rounds of PCR. iii) In the third step, the product of the second OEPR PCR round is digested with *DpnI* and then directly transformed into chemically competent *E. coli* TOP10F’ cells (Fig. 1C). The overhangs (homologous region A, Fig. 1C) can be repaired *in vivo* via the homologous recombination mechanism in *E. coli*.

**Figure 2:**
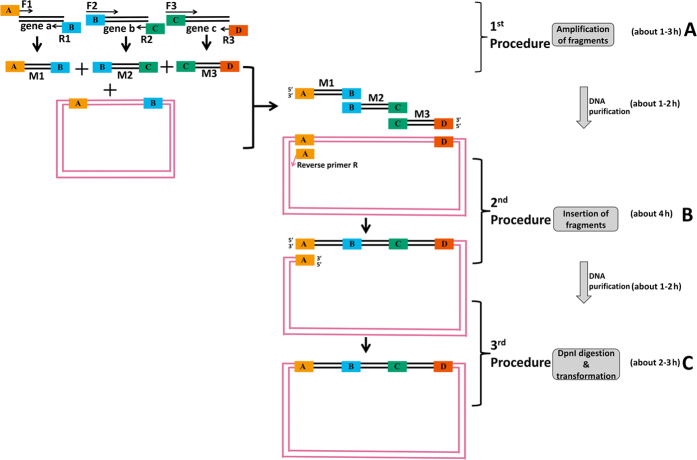
Schematic diagram and flowchart of OEPR cloning for insertion of multiple fragments. The cloning method requires three procedures: (**A**) amplification of insert fragments a, b and c with primers F1 and R1, F2 and R2 and F3 and R3, respectively; (**B**) exponential amplification using fragments (a, b, and c) generated in the first PCR and reverse primer R; and (**C**) digestion by *DpnI* to eliminate the parent DNA and direct transformation into chemically competent Top10F’ cells.

In addition, the mechanism of OEPR cloning for assembly of multi-fragments is shown in Fig. 2, by using the assembly of three inserts as an example. Three steps are similar to the OEPR cloning for insertion of single fragments. As shown in Fig. 2, the assembly of three fragments into the vector was used as an example. (i) In the first step, three genes (a, b and c) are amplified by using the primer pairs of F1 and R1, F2 and R2, and F3 and R3, respectively (Fig. 2A). (ii) In the second step, the 1^st^ PCR products (M1, M2 and M3) and the vector anneal with their overlapping homologous regions (parts B, C and D, Fig. 2B) and are exponentially amplified by product M1 and reverse primer R (Fig. 2B). (iii) The final linearized plamid with homologous overhangs (part A, Fig. 2C) in both 5′- and 3′-termini can be repaired *in vivo* via the homologous recombination mechanism in *E. coli* (Fig. 2C).

### Efficiency and fidelity of OEPR cloning with different overhangs

**Figure 3:**
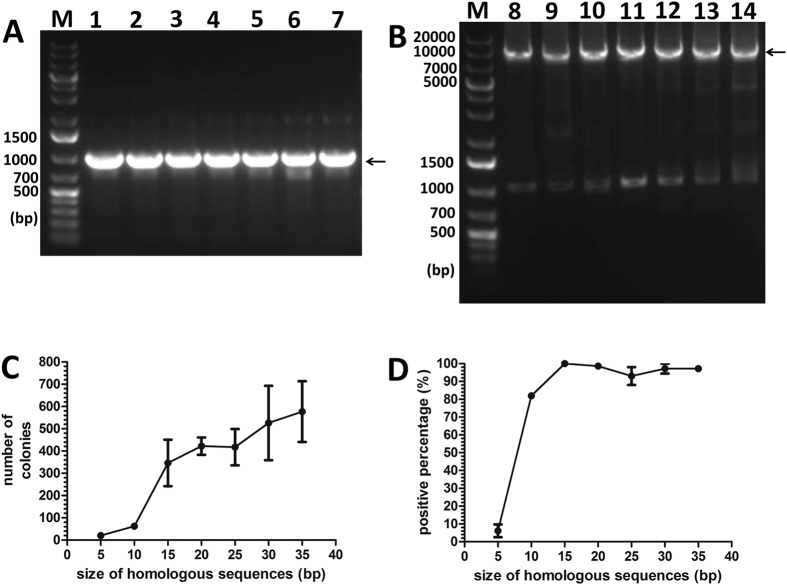
Efficiency and fidelity of OEPR cloning for different sizes of homology arms. (**A**) Different homology arms (5, 10, 15, 20, 25, 30 and 35 bp (lanes 1–7, respectively)) were added to the 1 kb fragment from hNa_V_1.5. PCR product were detected by agarose gel electrophoresis. The arrow indicates the target band of the first PCR. (**B**) The purified fragments (containing 5, 10, 15, 20, 25, 30 and 35 bp homology arms (lanes 8–14, respectively)) from the 1^st^ PCR were inserted into pGADT_7_ (7988 bp). PCR products were detected by agarose gel electrophoresis. The arrow indicates the target band of the second PCR. (**C**) Colony numbers per plate were counted to estimate cloning efficiencies. (**D**) The percentage of positive clones, which was estimated using colony PCR, was used to estimate cloning fidelities. Reported results are the mean ± SEM of three independent experiments. M: DNA molecular weight marker.

In the first series of experiments, we examined the effect of the end homology length of part A (Fig. 1) on OEPR cloning efficiency. A series of F1 primers containing different 5′ overhang lengths (5 to 35 bp) was used for the insertion of a 1 kb fragment into pGADT_7_ (7988 bp) (Fig. 3). Insert fragments (1 kb) were amplified in the first PCR (Fig. 3A), and the resulting products were used as primers for pGADT_7_ amplification (Fig. 3B). Obviously, more recombinant colonies (>300) and higher cloning efficiencies (>90%) were obtained with homology arms (part A in Fig. 1) longer than 15 bp (Fig. 3C,D). Interestingly, compared to other homologous region length groups, the group with the 15 bp homology region yielded the highest positive percentage (Fig. 3D, 100%).

### Insertion of long fragments of different sizes

**Figure 4:**
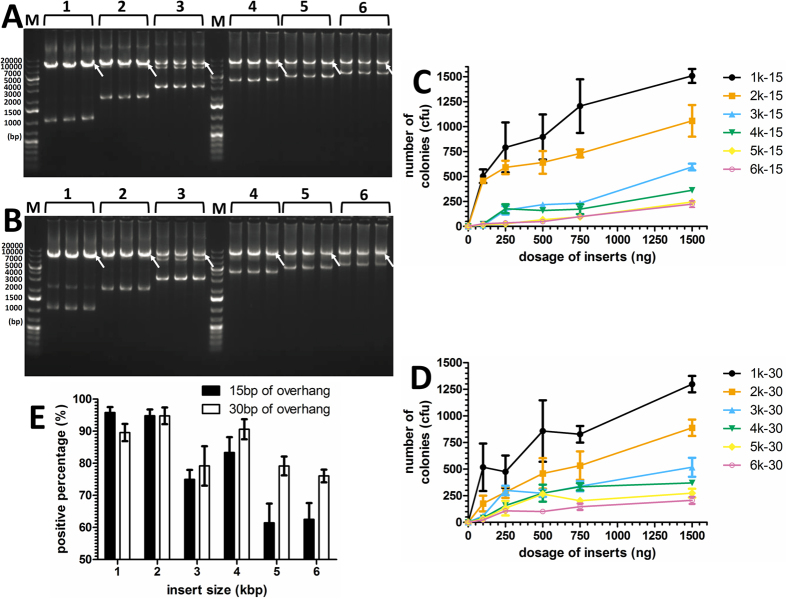
Large fragment insertion by OEPR cloning. (**A**) and (**B**) Large fragments of various lengths (1, 2, 3, 4, 5 and 6 kb (lanes 1–6, respectively)) containing 15 bp and 30 bp homology arms ((**A**) and (**B**), respectively) amplified from hNa_V_1.5 were used along with reverse primer R as primers for the second PCR step. The second PCR product (5 μl) was detected by agarose gel electrophoresis. The white arrow indicates the target band of the final product after the second of PCR. Different dosages of insert fragments using 15 bp (**C**) and 30 bp (**D**) homology arms in the first PCR were step were examined. Colony numbers on plates were counted to estimate cloning efficiencies. (**E**) Percentages of positive clones, which were estimated by colony PCR, were determined to estimate cloning fidelities. Reported results are the mean ± SEM of three independent experiments. M: DNA molecular weight marker.

We next investigated the influence of insert size (1~6 kb) using 15 or 30 bp overhangs. Each fragment was inserted into the vector pGADT_7_ (7988 bp). As shown in Fig. 4A and B, the target bands were successfully amplified. In addition, it was found that if more purified insert fragment was used as template in the second PCR, more colonies were obtained on plates (Fig. 4C,D). For the 1 kb and 2 kb inserts, 15 bp overhangs tended to yield more recombinant colonies and positive percentages than 30 bp overhangs. For inserts larger than 2 kb (3 kb~6 kb), 30 bp overhangs tended to yield more recombinant colonies and positive percentages than 15 bp overhangs (Fig. 4C,D,E). Increasing insert length tended to reduce the number of observed colonies and the positive percentage; however, this behavior can be partially addressed by using longer homology arms (Fig. 4E).

### Insertion of multiple fragments

The experiments described above demonstrated that OEPR cloning is an efficient and precise method for inserting large fragments into vectors. To develop OEPR cloning into a more powerful tool for molecular cloning, we investigated whether multiple DNA fragments could be simultaneously reassembled with a vector using OEPR cloning. Insert fragments of different lengths were tested (two 1 kb, two 2 kb, two 3 kb, three 1 kb, three 2 kb and four 1 kb fragments were inserted into pGADT_7_). Our OEPR cloning method performed these assemblies well.

**Figure 5:**
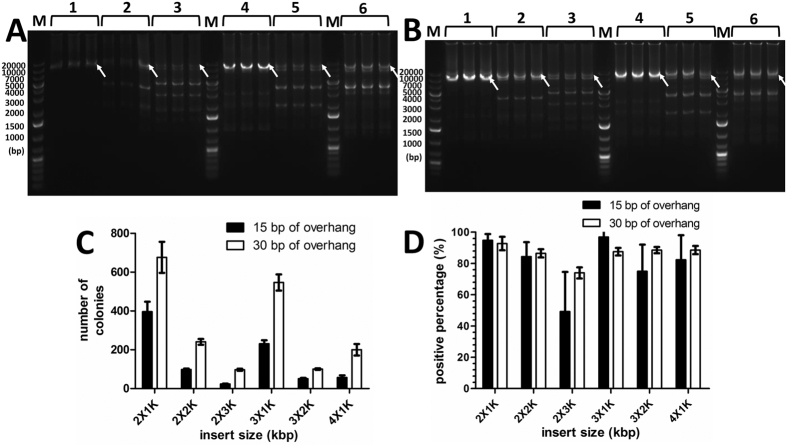
OEPR cloning for assembly of multiple DNA fragments. (**A**) and (**B**) Multiple fragments of different sizes (two 1 kb, two 2 kb, two 3 kb, three 1 kb, three 2 kb and four 1 kb (lanes 1–6, respectively)) as well as 15 bp and 30 bp homology arms ((**A**) and (**B**), respectively) amplified from rNa_V_1.4 and hNa_V_1.5 were used as primers along with reverse primer R in the second PCR step. The second PCR product (5 μl) was detected by agarose gel electrophoresis. The white arrow indicates the target band of the final product after the second of PCR. (**C**) Colony numbers per plates were determined to estimate cloning efficiencies for insertions of multiple fragments with 15 bp and 30 bp homology arms. (**D**) The percentages of positive clones, which were estimated by colony PCR, were used to estimate cloning fidelities. The results are the mean ± SEM of three independent experiments. M: DNA molecular weight marker.

More specifically, all fragments of interest were amplified in a first PCR step, then purified. Fragment a (500 ng) and the other fragments (100 ng each) were added to the second PCR as forward primer and templates, respectively. After the second PCR step, the final PCR product containing the desired homologous region (part A in Fig. 2) could be detected by agarose gel electrophoresis (Fig. 5A,B). It was shown that 30 bp overhangs yielded more recombinant colonies than 15 bp overhangs (Fig. 5C). With regard to positive percentages, the 15 bp overhangs were more suitable for the assembly of shorter and/or simple fragments (two 1 kb and three 1 kb of fragments), while 30 bp overhangs were preferable for the assembly of longer fragments (Fig. 5D).

## Discussion

Similar strategies of OEPR cloning have been reported previously, such as RF cloning^11^, CPEC (Circular Polymerase Extension Cloning) cloning^23^,^24^, EMP cloning^13^ and IFPC cloning^14^. In our opinions, all the methods of CPEC, EMP, IFPC and our OEPR cloning are derivatives of the RF cloning. In RF cloning, the amplified inserts are as long as the primers to amplify the vector, without adding extra sequences. Additionally, in CPEC method, a derivative method of RF cloning, the linear vector and inserts could anneal with their overlapping regions and extend using each other as a template. The most significant drawback of CPEC or RF is non-exponential amplification. Although, due to the introduction of the reverse primer, exponential amplification can be finished in both EMP and IFPC methods, the expensive enzymes [T4 PNK (T4 Polynucleotide Kinase) and T4 DNA ligase] are indispensable. But our OEPR cloning, with introduction of homologous regions of primer F1 of inserts with the vector (Figs 1A and 2A), the target products can be exponentially amplified *in vitro* and it can be directly transferred to *E. coli* without using expensive enzymes. So, OEPR cloning take the advantages of both RF cloning and EMP cloning, low cost and exponential amplification, respectively. Even using the home-made chemical competent cells with low transformation efficiency (about 10^6^ cfu/μg), the efficiency of OEPR cloning is still working well. In addition, the whole experiment of OEPR cloning, purification and transformation can be finished in one day (about 9–14 hours, Figs 1 and 2).

Overall, the most significant features of OEPR cloning are that it combines the advantages of both PCR-based strategies and recombinant enzyme-based methods and that it works well. OEPR cloning is an efficient, labor- and cost-saving strategy for the seamless insertion of large DNA fragments (up to 6 kb) or multiple DNA fragments (up to two 3 kb, three 2 kb and four 1 kb) into vectors (8 kb tested). In conclusion, OEPR cloning is a good choice for researchers who perform plasmid-based genetic manipulations such as gene mutation, gene chimeragenesis, and gene fusion.

## Methods

### Materials

*E. coli* TOP10F’ (Invitrogen, RecA- strain) was used for cloning. Chemically competent TOP10F’ cells were home-made (10^6^ cfu/μg). All insert fragments generated from hNa_V_1.5 and rNa_V_1.4 were inserted into the plasmid pGADT_7_ (Clontech, 7988 bp). All primers were purchased from Sangon (Shanghai, China). The insert, insertion site, and primer sequences are provided in the Supplementary Table S1 and S2.

### Design of primers for insertion of large fragments

In this part, the insertion of 1 kb of fragment into the vector was used as an example. As shown in Fig. 1, the target gene a (insert-1 in Supplementary Table S1) and vector are marked in black and reddish purple, respectively. The black parts of primers F1 (the non-underlined sequences of the primers of F1-15, F1-20, F1-25, F1-30 and F1-35 in Supplementary Table S2) and R1 (the non-underlined sequences of the primers of 1kb-R1 in Supplementary Table S2) are homologous with the target gene and have Tm values of approximately 68–70 °C. The orange part of primer F1 is a 15–30 bp vector-derived homology region (the underlined parts of the primers of F1-15, F1-20, F1-25 and F1-30 in Supplementary Table S2). The sky blue part of primer R1 (the underlined sequences of the primers of 1kb-R1 in Supplementary Table S2) is homologous with the vector with a Tm of approximately 68–70 °C.

### Design of primers for assembly of multiple fragments

In this part, the assembly of three 1 kb of fragments (insert-10 in Supplementary Table S1) into the vector was used as an example. As shown in Fig. 2, three insert genes a, b and c, (the parts of insert-10 are shaded in yellow, azure and yellow respectively in Supplementary Table S1) are amplified using primer pairs F1 and R1, F2 and R2 and F3 and R3 (the primer pairs of 3 × 1k-F1-15/30 and 3 × 1k-R1, 3 × 1k-F2 and 3 × 1k-R2, 3 × 1k-F3 and 3 × 1k-R3 respectively in Supplementary Table S2), respectively in Fig. 2A. The target gene and the vector are marked in black and reddish purple in Fig. 2, respectively. The black parts of the primers F1, R1, F2, R2, F3 and R3 (the bold sequences of the primers of 3 × 1k-F1-15/30, 3 × 1k-R1, 3 × 1k-F2, 3 × 1k-R2, 3 × 1k-F3 and 3 × 1k-R3 in Supplementary Table S2) are homologous with the target gene and have a Tm of approximately 68–70 °C. The orange part of primer F1 (the underlined sequences of the primers of 3 × 1k-F1-15/30 in Supplementary Table S2) is a 15–30 bp vector-derived homology region. The sky blue, bluish green and vermilion parts (part B, C and D, respectively) (the italic sequences of the primers of 3 × 1k-R1, 3 × 1k-R2 and the underlined sequences of primer 3 × 1k-R3 in Supplementary Table S2) of primers R1, R2 and R3 are homologous with gene b, gene c and the vector insertion site, respectively, with Tm values of approximately 68–70 °C.

### Synthesis of large inserts

DNA fragments of different sizes (1~6 kb) were amplified from hNa_V_1.5 using KOD-FX (TOYOBO) and 0.2 μM of each primer, 1 × reaction buffer, 0.4 mM dNTPs, 1 U DNA polymerase and 10 ng template DNA in 50 μl reaction mixtures. Mixtures were first denatured at 94 °C for 2 min, subjected to 25 cycles of denaturation at 98 °C for 10 s, annealing at 60 °C for 30 s and elongation at 68 °C for 25 cycles (1 kb/min), followed by a final 10 min extension step at 68 °C. PCR products were analyzed by agarose gel electrophoresis, and target fragments were purified using a DNA purification Kit (BioSci, Hangzhou, China).

### Insertion of large fragments

Fragments purified from the first PCR step and the reverse primer R were used to exponentially amplify the plasmid pGADT_7_ (as shown in Fig. 1). The PCR mixture contained 0.2 μM primers, 10 ng recipient plasmid, 1 × PCR Buffer, 0.4 mM dNTPs and 1 U DNA polymerase in a 50 μl reaction mixture. Mixtures were pre-denatured at 94 °C for 2 min, subjected to 25 cycles of denaturation at 98 °C for 10 s, annealing at 60 °C for 30 s and elongation at 68 °C for 25 cycles (1 kb/min), followed by a final 10 min extension step at 68 °C. The resulting PCR products were purified using a DNA purification Kit (BioSci, Hangzhou, China). Following purification, PCR products were digested using *DpnI* as follows: purified products (8 μl) supplemented with 1 μl 10 × reaction buffer were digested with 1 μl FastDigest^TM^ *DpnI* (Thermo Fermentas, Burlington, Canada) at 37 °C for 1 h.

### Insertion of multiple fragments

As shown in Fig. 2, OEPR cloning can also be used to simultaneously insert multiple DNA fragments. First, each insert fragment (e.g., genes a, b and c, Fig. 2A) is amplified using KOD-FX and purified using a DNA purification kit (BioSci, Hangzhou, China). Second, 500 ng of purified fragment M1 (which is used as the forward primer) and 100 ng of the other fragments (M2 and M3, shown in Fig. 2B) are added with reverse primer R into a PCR mixture for exponential vector amplification (shown in Fig. 2B). 5′ ends of the inserts contains 15 or 30 nt of homology to the vector. Reverse primer R is homologous to the vector with a Tm of approximately 68–70 °C. The inserts (M1, M2 and M3) and the vector anneal with their overlapping homologous regions (parts B, C and D, Fig. 2B) and are exponentially amplified by product M1 and reverse primer R. After this second PCR step, linear products with homologous region A (Fig. 2C, indicated in orange), which can be directly transformed and repaired by *E. coli in vivo* (RecA- strain), are obtained. DNA products from the second PCR were purified using a DNA purification kit (BioSci, Hangzhou, China). Purified products (8 μl) were supplemented with 1 μl 10 × reaction buffer and digested using 1 μl FastDigest^TM^ *DpnI* (Thermo Fermentas, Burlington, Canada) at 37 °C for 1 h.

### Transformation, colony PCR and DNA sequencing

Aliquots (5 μl) of digested DNA products were directly transformed into 50 μl of chemically competent TOP10F’ cells that were made in-house. All cells were spread onto Luria-Bertani (LB) plates containing 100 μg/mL of ampicillin sodium salt and incubated overnight. Colony numbers per plate were determined to estimate cloning efficiencies. Colony PCR was used to screen 24 colonies randomly picked from each group. The percentages of positive clones obtained via colony PCR were used to estimate cloning fidelity. Colony PCR was performed using DreamTaq DNA polymerase (Thermo Scientific, USA) according to the manufacturer’s instructions. Further DNA sequencing was performed by Sangon (Shanghai, China).

## Additional Information

**How to cite this article**: Liu, C.-J. *et al*. OEPR Cloning: an Efficient and Seamless Cloning Strategy for Large- and Multi-Fragments. *Sci. Rep.* **7**, 44648; doi: 10.1038/srep44648 (2017).

**Publisher's note:** Springer Nature remains neutral with regard to jurisdictional claims in published maps and institutional affiliations.

## Supplementary Material

Supplementary Tables
