# Supplementary material for: OEPR Cloning: an Efficient and Seamless Cloning Strategy for Large- and Multi-Fragments
Source: Sci Rep. 2017 Mar 16;7:44648. doi: 10.1038/srep44648 (PMC5353728; doi:10.1038/srep44648)
Supplement: Supplementary Tables [file srep44648-s1.pdf]

Supplementary Information

OEPR Cloning: an Efficient and Seamless Cloning Strategy for Large- and Multi-Fragments

Chang-Jun Liu [1,†], Hui Jiang [2,†], Lei Wu [1], Ling-Yun Zhu [1], Er Meng [1,*] and Dong-Yi Zhang [1,*]

[1] Research Center of Biological Information, College of Science, National University of Defense Technology, Changsha, Hunan 410073, China

[2] Beijing Institute of Pharmaceutical Chemistry, Beijing 102205, China

* To whom correspondence should be addressed. E-mail: dongyizhang@nudt.edu.cn or er_meng@qq.com

[†]These authors contributed equally to this work as first authors.

Supplementary Table S1. Nucleic acid sequences of inserts and the insertion site of inserts into vector $pGADT_7$.

Name	Size of inserts	Sequences
Insert-1	1 kb	ATGGCAAACTTCCTATTACCTCGGGGCACCAGCAGCTTCCGC<br>AGGTTCACACGGGAGTCCCTGGCAGCCATCGAGAAGCGCATG<br>GCGGAGAAGCAAGCCCGCGGCTCAACCACCTTGCAGGAGAGC<br>CGAGAGGGGCTGCCCGAGGAGGAGGCTCCCCGGCCCCAGCTG<br>GACCTGCAGGCCTCCAAAAAGCTGCCAGATCTCTATGGCAAT<br>CCACCCCAAGAGCTCATCGGAGAGCCCCTGGAGGACCTGGAC<br>CCCTTCTATAGCACCCAAAAGACTTTCATCGTACTGAATAAA<br>GGCAAGACCATCTTCCGGTTCAGTGCCACCAACGCCTTGTAT<br>GTCCTCAGTCCCTTCCACCCCATCCGGAGAGCGGCTGTGAAG<br>ATTCTGGTTCACTCGCTCTTCAACATGCTCATCATGTGCACC<br>ATCCTCACCAACTGCGTGTTCATGGCCCAGCACGACCCTCCA<br>CCCTGGACCAAGTATGTCGAGTACACCTTCACCGCCATTTAC<br>ACCTTTGAGTCTCTGGTCAAGATTCTGGCTCGAGGCTTCTGC<br>CTGCACGCGTTCACTTTCCTTCGGGACCCATGGAACTGGCTG<br>GACTTTAGTGTGATTATCATGGCATACACAACTGAATTTGTG<br>GACCTGGGCAATGTCTCAGCCTTACGCACCTTCCGAGTCCTC<br>CGGGCCCTGAAAACTATATCAGTCATTTCAGGGCTGAAGACC<br>ATCGTGGGGGCCCTGATCCAGTCTGTGAAGAAGCTGGCTGAT<br>GTGATGGTCCTCACAGTCTTCTGCCTCAGCGTCTTTGCCCTC<br>ATCGGCCTGCAGCTCTTCATGGGCAACCTAAGGCACAAGTGC<br>GTGCGCAACTTCACAGCGCTCAACGGCACCAACGGCTCCGTG<br>GAGGCCGACGGCTTGGTCTGGGAATCCCTGGACCTTTACCTC<br>AGTGATCCAGAAAATTACCTGCTCAAGAACGGCACCTCTGAT<br>GTGTTACTGTGTGGGAACAGCTCTGACGCTGGGA
Insert-2	2 kb	ATGGCAAACTTCCTATTACCTCGGGGCACCAGCAGCTTCCGC<br>AGGTTCACACGGGAGTCCCTGGCAGCCATCGAGAAGCGCATG<br>GCGGAGAAGCAAGCCCGCGGCTCAACCACCTTGCAGGAGAGC<br>CGAGAGGGGCTGCCCGAGGAGGAGGCTCCCCGGCCCCAGCTG<br>GACCTGCAGGCCTCCAAAAAGCTGCCAGATCTCTATGGCAAT<br>CCACCCCAAGAGCTCATCGGAGAGCCCCTGGAGGACCTGGAC<br>CCCTTCTATAGCACCCAAAAGACTTTCATCGTACTGAATAAA<br>GGCAAGACCATCTTCCGGTTCAGTGCCACCAACGCCTTGTAT<br>GTCCTCAGTCCCTTCCACCCCATCCGGAGAGCGGCTGTGAAG<br>ATTCTGGTTCACTCGCTCTTCAACATGCTCATCATGTGCACC<br>ATCCTCACCAACTGCGTGTTCATGGCCCAGCACGACCCTCCA<br>CCCTGGACCAAGTATGTCGAGTACACCTTCACCGCCATTTAC<br>ACCTTTGAGTCTCTGGTCAAGATTCTGGCTCGAGGCTTCTGC<br>CTGCACGCGTTCACTTTCCTTCGGGACCCATGGAACTGGCTG<br>GACTTTAGTGTGATTATCATGGCATACACAACTGAATTTGTG<br>GACCTGGGCAATGTCTCAGCCTTACGCACCTTCCGAGTCCTC

		CGGGCCCTGAAAACTATATCAGTCATTTCAGGGCTGAAGACC<br>ATCGTGGGGGCCCTGATCCAGTCTGTGAAGAAGCTGGCTGAT<br>GTGATGGTCCTCACAGTCTTCTGCCTCAGCGTCTTTGCCCTC<br>ATCGGCCTGCAGCTCTTCATGGGCAACCTAAGGCACAAGTGC<br>GTGCGCAACTTCACAGCGCTCAACGGCACCAACGGCTCCGTG<br>GAGGCCGACGGCTTGGTCTGGGAATCCCTGGACCTTTACCTC<br>AGTGATCCAGAAAATTACCTGCTCAAGAACGGCACCTCTGAT<br>GTGTTACTGTGTGGGAACAGCTCTGACGCTGGGACATGTCCG<br>GAGGGCTACCGGTGCCTAAAGGCAGGCGAGAACCCCGACCAC<br>GGCTACACCAGCTTCGATTCCTTTGCCTGGGCCTTTCTTGCA<br>CTCTTCCGCCTGATGACGCAGGACTGCTGGGAGCGCCTCTAT<br>CAGCAGACCCTCAGGTCCGCAGGGAAGATCTACATGATCTTC<br>TTCATGCTTGTCATCTTCCTGGGGTCCTTCTACCTGGTGAAC<br>CTGATCCTGGCCGTGGTCGCAATGGCCTATGAGGAGCAAAAC<br>CAAGCCACCATCGCTGAGACCGAGGAGAAGGAAAAGCGCTTC<br>CAGGAGGCCATGGAAATGCTCAAGAAAGAACACGAGGCCCTC<br>ACCATCAGGGGTGTGGATACCGTGTCCCGTAGCTCCTTGGAG<br>ATGTCCCCTTTGGCCCCAGTAAACAGCCATGAGAGAAGAAGC<br>AAGAGGAGAAAACGGATGTCTTCAGGAACTGAGGAGTGTGGG<br>GAGGACAGGCTCCCCAAGTCTGACTCAGAAGATGGTCCCAGA<br>GCAATGAATCATCTCAGCCTCACCCGTGGCCTCAGCAGGACT<br>TCTATGAAGCCACGTTCCAGCCGCGGGAGCATTTTCACCTTT<br>CGCAGGCGAGACCTGGGTTCTGAAGCAGATTTTGCAGATGAT<br>GAAAACAGCACAGCGGGGGGAGAGCGAGAGCCACCACGCATC<br>ACTGCTGGTGCCCTGGCCCCTGCGCCGGACCAGTGCCCAGGG<br>ACAGCCCAGTCCCGGAACCTCGGCTCCTGGCCACGCCCTCCA<br>TGGCAAAAAGAACAGCACTGTGGACTGCAATGGGGTGGTCTC<br>ATTACTGGGGGCAGGCGACCCAGAGGCCACATCCCCAGGAAG<br>CCACCTCCTCCGCCCTGTGATGCTAGAGCACCCGCCAGACAC<br>GACCACGCCATCGGAGGAGCCAGGCGGGCCCCAGATGCTGAC<br>CTCCCAGGCTCCGTGTGTAGATGGCTTCGAGGAGCCAGGAGC<br>ACGGCAGCGGGCCCTCAGCGCAGTCA
Insert-3	3 kb	ATGGCAAACTTCCTATTACCTCGGGGCACCAGCAGCTTCCGC<br>AGGTTCACACGGGAGTCCCTGGCAGCCATCGAGAAGCGCATG<br>GCGGAGAAGCAAGCCCGCGGCTCAACCACCTTGCAGGAGAGC<br>CGAGAGGGGCTGCCCGAGGAGGAGGCTCCCCGGCCCCAGCTG<br>GACCTGCAGGCCTCCAAAAAGCTGCCAGATCTCTATGGCAAT<br>CCACCCCAAGAGCTCATCGGAGAGCCCCTGGAGGACCTGGAC<br>CCCTTCTATAGCACCCAAAAGACTTTCATCGTACTGAATAAA<br>GGCAAGACCATCTTCCGGTTCAGTGCCACCAACGCCTTGTAT<br>GTCCTCAGTCCCTTCCACCCCATCCGGAGAGCGGCTGTGAAG<br>ATTCTGGTTCACTCGCTCTTCAACATGCTCATCATGTGCACC<br>ATCCTCACCAACTGCGTGTTCATGGCCCAGCACGACCCTCCA<br>CCCTGGACCAAGTATGTCGAGTACACCTTCACCGCCATTTAC

		ACCTTTGAGTCTCTGGTCAAGATTCTGGCTCGAGGCTTCTGC<br>CTGCACGCGTTCACTTTCCTTCGGGACCCATGGAACTGGCTG<br>GACTTTAGTGTGATTATCATGGCATACACAACTGAATTTGTG<br>GACCTGGGCAATGTCTCAGCCTTACGCACCTTCCGAGTCCTC<br>CGGGCCCTGAAAACTATATCAGTCATTTCAGGGCTGAAGACC<br>ATCGTGGGGGCCCTGATCCAGTCTGTGAAGAAGCTGGCTGAT<br>GTGATGGTCCTCACAGTCTTCTGCCTCAGCGTCTTTGCCCTC<br>ATCGGCCTGCAGCTCTTCATGGGCAACCTAAGGCACAAGTGC<br>GTGCGCAACTTCACAGCGCTCAACGGCACCAACGGCTCCGTG<br>GAGGCCGACGGCTTGGTCTGGGAATCCCTGGACCTTTACCTC<br>AGTGATCCAGAAAATTACCTGCTCAAGAACGGCACCTCTGAT<br>GTGTTACTGTGTGGGAACAGCTCTGACGCTGGGACATGTCCG<br>GAGGGCTACCGGTGCCTAAAGGCAGGCGAGAACCCCGACCAC<br>GGCTACACCAGCTTCGATTCCTTTGCCTGGGCCTTTCTTGCA<br>CTCTTCCGCCTGATGACGCAGGACTGCTGGGAGCGCCTCTAT<br>CAGCAGACCCTCAGGTCCGCAGGGAAGATCTACATGATCTTC<br>TTCATGCTTGTCATCTTCCTGGGGTCCTTCTACCTGGTGAAC<br>CTGATCCTGGCCGTGGTCGCAATGGCCTATGAGGAGCAAAAC<br>CAAGCCACCATCGCTGAGACCGAGGAGAAGGAAAAGCGCTTC<br>CAGGAGGCCATGGAAATGCTCAAGAAAGAACACGAGGCCCTC<br>ACCATCAGGGGTGTGGATACCGTGTCCCGTAGCTCCTTGGAG<br>ATGTCCCCTTTGGCCCCAGTAAACAGCCATGAGAGAAGAAGC<br>AAGAGGAGAAAACGGATGTCTTCAGGAACTGAGGAGTGTGGG<br>GAGGACAGGCTCCCCAAGTCTGACTCAGAAGATGGTCCCAGA<br>GCAATGAATCATCTCAGCCTCACCCGTGGCCTCAGCAGGACT<br>TCTATGAAGCCACGTTCCAGCCGCGGGAGCATTTTCACCTTT<br>CGCAGGCGAGACCTGGGTTCTGAAGCAGATTTTGCAGATGAT<br>GAAAACAGCACAGCGGGGGAGAGCGAGAGCCACCACGCATC<br>ACTGCTGGTGCCCTGGCCCCTGCGCCGGACCAGTGCCCAGGG<br>ACAGCCCAGTCCCGGAACCTCGGCTCCTGGCCACGCCCTCCA<br>TGGCAAAAAGAACAGCACTGTGGACTGCAATGGGGTGGTCTC<br>ATTACTGGGGGCAGGCGACCCAGAGGCCACATCCCCAGGAAG<br>CCACCTCCTCCGCCCTGTGATGCTAGAGCACCCGCCAGACAC<br>GACCACGCCATCGGAGGAGCCAGGCGGGCCCCAGATGCTGAC<br>CTCCCAGGCTCCGTGTGTAGATGGCTTCGAGGAGCCAGGAGC<br>ACGGCAGCGGGCCCTCAGCGCAGTCAGCGTCCTCACCAGCGC<br>ACTGGAAGAGTTAGAGGAGTCTCGCCATAAGTGTCCACCATG<br>CTGGAACCGTCTCGCCCAGCGCTACCTGATCTGGGAGTGCTG<br>CCCGCTGTGGATGTCCATCAAGCAGGGAGTGAAGTTGGTGGT<br>CATGGACCCGTTTACTGACCTCACCATCACTATGTGCATCGT<br>ACTCAACACACTCTTCATGGCGCTGGAGCACTACAACATGAC<br>AAGTGAATTCGAGGAGATGCTGCAGGTCGGAAACCTGGTCTT<br>CACAGGGATTTTCACAGCAGAGATGACCTTCAAGATCATTGC<br>CCTCGACCCCTACTACTACTTCCAACAGGGCTGGAACATCTT

		CGACAGCATCATCGTCATCCTTAGCCTCATGGAGCTGGGCCT<br>GTCCCGCATGAGCAACTTGTCGGTGCTGCGCTCCTTCCGCCT<br>GCTGCGGGTCTTCAAGCTGGCCAAATCATGGCCCACCCTGAA<br>CACACTCATCAAGATCATCGGGAACTCAGTGGGGGCACTGGG<br>GAACCTGACACTGGTGCTAGCCATCATCGTGTTCATCTTTGC<br>TGTGGTGGGCATGCAGCTCTTTGGCAAGAACTACTCGGAGCT<br>GAGGGACAGCGACTCAGGCCTGCTGCCTCGCTGGCACATGAT<br>GGACTTCTTTCATGCCTTCCTCATCATCTTCCGCATCCTCTG<br>TGGAGAGTGGATCGAGACCATGTGGGACTGCATGGAGGTGTC<br>GGGGCAGTCATTATGCCTGCTGGTCTTCTTGCTTGTTATGGT<br>CATTGGCAACCTTGTGGTCCTGAATCTCTTCCTGGCCTTGCT<br>GCTCAGCTCCTTCAGTGCAGACAACCTCACAGCCCCTGATGA<br>GGACAGAGAAGATGAACAACCTCCAAGCTGGCCCTGGCCCGC<br>ATCCAGAAGGGGCCTGCGCTTTGTCAAGCGGACCACCTGGGA<br>TTTCTGCTGTGGTCTCCTGCGGCAGCGGCCTCAGAAGCCCGC<br>AGCCCTTGCCGC
Insert-4	4 kb	ATGGCAAACTTCCTATTACCTCGGGGCACCAGCAGCTTCCGC<br>AGGTTCACACGGGAGTCCCTGGCAGCCATCGAGAAGCGCATG<br>GCGGAGAAGCAAGCCCGCGGCTCAACCACCTTGCAGGAGAGC<br>CGAGAGGGGCTGCCCGAGGAGGAGGCTCCCCGGCCCCAGCTG<br>GACCTGCAGGCCTCCAAAAAGCTGCCAGATCTCTATGGCAAT<br>CCACCCCAAGAGCTCATCGGAGAGCCCCTGGAGGACCTGGAC<br>CCCTTCTATAGCACCCAAAAGACTTTCATCGTACTGAATAAA<br>GGCAAGACCATCTTCCGGTTCAGTGCCACCAACGCCTTGTAT<br>GTCCTCAGTCCCTTCCACCCCATCCGGAGAGCGGCTGTGAAG<br>ATTCTGGTTCACTCGCTCTTCAACATGCTCATCATGTGCACC<br>ATCCTCACCAACTGCGTGTTCATGGCCCAGCACGACCCTCCA<br>CCCTGGACCAAGTATGTCGAGTACACCTTCACCGCCATTTAC<br>ACCTTTGAGTCTCTGGTCAAGATTCTGGCTCGAGGCTTCTGC<br>CTGCACGCGTTCACTTTCCTTCGGGACCCATGGAACTGGCTG<br>GACTTTAGTGTGATTATCATGGCATACACAACTGAATTTGTG<br>GACCTGGGCAATGTCTCAGCCTTACGCACCTTCCGAGTCCTC<br>CGGGCCCTGAAAACTATATCAGTCATTTCAGGGCTGAAGACC<br>ATCGTGGGGGCCCTGATCCAGTCTGTGAAGAAGCTGGCTGAT<br>GTGATGGTCCTCACAGTCTTCTGCCTCAGCGTCTTTGCCCTC<br>ATCGGCCTGCAGCTCTTCATGGGCAACCTAAGGCACAAGTGC<br>GTGCGCAACTTCACAGCGCTCAACGGCACCAACGGCTCCGTG<br>GAGGCCGACGGCTTGGTCTGGGAATCCCTGGACCTTTACCTC<br>AGTGATCCAGAAAATTACCTGCTCAAGAACGGCACCTCTGAT<br>GTGTTACTGTGTGGGAACAGCTCTGACGCTGGGACATGTCCG<br>GAGGGCTACCGGTGCCTAAAGGCAGGCGAGAACCCCGACCAC<br>GGCTACACCAGCTTCGATTCCTTTGCCTGGGCCTTTCTTGCA<br>CTCTTCCGCCTGATGACGCAGGACTGCTGGGAGCGCCTCTAT<br>CAGCAGACCCTCAGGTCCGCAGGGAAGATCTACATGATCTTC

		TTCATGCTTGTCATCTTCCTGGGGTCCTTCTACCTGGTGAAC<br>CTGATCCTGGCCGTGGTCGCAATGGCCTATGAGGAGCAAAAC<br>CAAGCCACCATCGCTGAGACCGAGGAGAAGGAAAAGCGCTTC<br>CAGGAGGCCATGGAAATGCTCAAGAAAGAACACGAGGCCCTC<br>ACCATCAGGGGTGTGGATACCGTGTCCCGTAGCTCCTTGGAG<br>ATGTCCCCTTTGGCCCCAGTAAACAGCCATGAGAGAAGAAGC<br>AAGAGGAGAAAACGGATGTCTTCAGGAACTGAGGAGTGTGGG<br>GAGGACAGGCTCCCCAAGTCTGACTCAGAAGATGGTCCCAGA<br>GCAATGAATCATCTCAGCCTCACCCGTGGCCTCAGCAGGACT<br>TCTATGAAGCCACGTTCCAGCCGCGGGAGCATTTTCACCTTT<br>CGCAGGCGAGACCTGGGTTCTGAAGCAGATTTTGCAGATGAT<br>GAAAACAGCACAGCGGGGGGAGAGCGAGAGCCACCACGCATC<br>ACTGCTGGTGCCCTGGCCCCTGCGCCGGACCAGTGCCCAGGG<br>ACAGCCCAGTCCCGGAACCTCGGCTCCTGGCCACGCCCTCCA<br>TGGCAAAAAGAACAGCACTGTGGACTGCAATGGGGTGGTCTC<br>ATTACTGGGGGCAGGCGACCCAGAGGCCACATCCCCAGGAAG<br>CCACCTCCTCCGCCCTGTGATGCTAGAGCACCCGCCAGACAC<br>GACCACGCCATCGGAGGAGCCAGGCGGGCCCCAGATGCTGAC<br>CTCCCAGGCTCCGTGTGTAGATGGCTTCGAGGAGCCAGGAGC<br>ACGGCAGCGGGCCCTCAGCGCAGTCAGCGTCCTCACCAGCGC<br>ACTGGAAGAGTTAGAGGAGTCTCGCCATAAGTGTCCACCATG<br>CTGGAACCGTCTCGCCCAGCGCTACCTGATCTGGGAGTGCTG<br>CCCGCTGTGGATGTCCATCAAGCAGGGAGTGAAGTTGGTGGT<br>CATGGACCCGTTTACTGACCTCACCATCACTATGTGCATCGT<br>ACTCAACACACTCTTCATGGCGCTGGAGCACTACAACATGAC<br>AAGTGAATTCGAGGAGATGCTGCAGGTCGGAAACCTGGTCTT<br>CACAGGGATTTTCACAGCAGAGATGACCTTCAAGATCATTGC<br>CCTCGACCCCTACTACTACTTCCAACAGGGCTGGAACATCTT<br>CGACAGCATCATCGTCATCCTTAGCCTCATGGAGCTGGGCCT<br>GTCCCGCATGAGCAACTTGTCGGTGCTGCGCTCCTTCCGCCT<br>GCTGCGGGTCTTCAAGCTGGCCAAATCATGGCCCACCCTGAA<br>CACACTCATCAAGATCATCGGGAACTCAGTGGGGGCACTGGG<br>GAACCTGACACTGGTGCTAGCCATCATCGTGTTCATCTTTGC<br>TGTGGTGGGCATGCAGCTCTTTGGCAAGAACTACTCGGAGCT<br>GAGGGACAGCGACTCAGGCCTGCTGCCTCGCTGGCACATGAT<br>GGACTTCTTTCATGCCTTCCTCATCATCTTCCGCATCCTCTG<br>TGGAGAGTGGATCGAGACCATGTGGGACTGCATGGAGGTGTC<br>GGGGCAGTCATTATGCCTGCTGGTCTTCTTGCTTGTTATGGT<br>CATTGGCAACCTTGTGGTCCTGAATCTCTTCCTGGCCTTGCT<br>GCTCAGCTCCTTCAGTGCAGACAACCTCACAGCCCCTGATGA<br>GGACAGAGAAGATGAACAACCTCCAAGCTGGCCCTGGCCCGC<br>ATCCAGAAGGGGCCTGCGCTTTGTCAAGCGGACCACCTGGGA<br>TTTCTGCTGTGGTCTCCTGCGGCAGCGGCCTCAGAAGCCCGC<br>AGCCCTTGCCGCCCAGGGCCAGCTGCCCAGCTGCATTGCCAC

		CCCCTACTCCCCGCCACCCCCAGAGACGGAGAAGGTGCCTCC<br>CACCCGCAAGGAAACACGGTTTGAGGAAGGCGAGCAACCAGG<br>CCAGGGCACCCCCGGGGATCCAGAGCCCGTGTGTGTGCCCAT<br>CGCTGTGGCCGAGTCAGACACAGATGACCAAGAAGAAGATGA<br>GGAGAACAGCCTGGGCACGGAGGAGGAGTCCAGCAAGCAGGA<br>ATCCCAGCCTGTGTCCGGTGGCCCAGAGGCCCCTCCGGATTC<br>CAGGACCTGGAGCCAGGTGTCAGCGACTGCCTCCTCTGAGGC<br>CGAGGCCAGTGCATCTCAGGCCGACTGGCGGCAGCAGTGGAA<br>AGCGGAACCCCAGGCCCCAGGGTGCGGTGAGACCCCAGAGGA<br>CAGTTGCTCCGAGGGCAGCACAGCAGACATGACCAACACCGC<br>TGAGCTCCTGGAGCAGATCCCTGACCTCGGCCAGGATGTCAA<br>GGACCCAGAGGACTGCTTCACTGAAGGCTGTGTCCGGCGCTG<br>TCCCTGCTGTGCGGTGGACACCACACAGGCCCCAGGGAAGGT<br>CTGGTGGCGGTTGCGCAAGACCTGCTACCACATCGTGGAGCA<br>CAGCTGGTTCGAGACATTCATCATCTTCATGATCCTACTCAG<br>CAGTGGAGCGCTGGCCTTCGAGGACATCTACCTAGAGGAGCG<br>GAAGACCATCAAGGTTCTGCTTGAGTATGCCGACAAGATGTT<br>CACATATGTCTTCGTGCTGGAGATGCTGCTCAAGTGGGTGGC<br>CTACGGCTTCAAGAAGTACTTCACCAATGCCTGGTGCTGGCT<br>CGACTTCCTCATCGTAGACGTCTCTCTGGTCAGCCTGGTGGC<br>CAACACCCTGGGCTTTGCCGAGATGGGCCCCATCAAGTCACT<br>GCGGACGCTGCGTGCACTCCGTCCTCTGAGAGCTCTGTCACG<br>ATTTGAGGGCATGAGGGTGGTGGTCAATGCCCTGGTGGGCGC<br>CA
Insert-5	5 kb	ATGGCAAACTTCCTATTACCTCGGGGCACCAGCAGCTTCCGC<br>AGGTTCACACGGGAGTCCCTGGCAGCCATCGAGAAGCGCATG<br>GCGGAGAAGCAAGCCCGCGGCTCAACCACCTTGCAGGAGAGC<br>CGAGAGGGGCTGCCCGAGGAGGAGGCTCCCCGGCCCCAGCTG<br>GACCTGCAGGCCTCCAAAAAGCTGCCAGATCTCTATGGCAAT<br>CCACCCCAAGAGCTCATCGGAGAGCCCCTGGAGGACCTGGAC<br>CCCTTCTATAGCACCCAAAAGACTTTCATCGTACTGAATAAA<br>GGCAAGACCATCTTCCGGTTCAGTGCCACCAACGCCTTGTAT<br>GTCCTCAGTCCCTTCCACCCCATCCGGAGAGCGGCTGTGAAG<br>ATTCTGGTTCACTCGCTCTTCAACATGCTCATCATGTGCACC<br>ATCCTCACCAACTGCGTGTTCATGGCCCAGCACGACCCTCCA<br>CCCTGGACCAAGTATGTCGAGTACACCTTCACCGCCATTTAC<br>ACCTTTGAGTCTCTGGTCAAGATTCTGGCTCGAGGCTTCTGC<br>CTGCACGCGTTCACTTTCCTTCGGGACCCATGGAACTGGCTG<br>GACTTTAGTGTGATTATCATGGCATACACAACTGAATTTGTG<br>GACCTGGGCAATGTCTCAGCCTTACGCACCTTCCGAGTCCTC<br>CGGGCCCTGAAAACTATATCAGTCATTTCAGGGCTGAAGACC<br>ATCGTGGGGGCCCTGATCCAGTCTGTGAAGAAGCTGGCTGAT<br>GTGATGGTCCTCACAGTCTTCTGCCTCAGCGTCTTTGCCCTC<br>ATCGGCCTGCAGCTCTTCATGGGCAACCTAAGGCACAAGTGC

		GTGCGCAACTTCACAGCGCTCAACGGCACCAACGGCTCCGTG<br>GAGGCCGACGGCTTGGTCTGGGAATCCCTGGACCTTTACCTC<br>AGTGATCCAGAAAATTACCTGCTCAAGAACGGCACCTCTGAT<br>GTGTTACTGTGTGGGAACAGCTCTGACGCTGGGACATGTCCG<br>GAGGGCTACCGGTGCCTAAAGGCAGGCGAGAACCCCGACCAC<br>GGCTACACCAGCTTCGATTCCTTTGCCTGGGCCTTTCTTGCA<br>CTCTTCCGCCTGATGACGCAGGACTGCTGGGAGCGCCTCTAT<br>CAGCAGACCCTCAGGTCCGCAGGGAAGATCTACATGATCTTC<br>TTCATGCTTGTCATCTTCCTGGGGTCCTTCTACCTGGTGAAC<br>CTGATCCTGGCCGTGGTCGCAATGGCCTATGAGGAGCAAAAC<br>CAAGCCACCATCGCTGAGACCGAGGAGAAGGAAAAGCGCTTC<br>CAGGAGGCCATGGAAATGCTCAAGAAAGAACACGAGGCCCTC<br>ACCATCAGGGGTGTGGATACCGTGTCCCGTAGCTCCTTGGAG<br>ATGTCCCCTTTGGCCCCAGTAAACAGCCATGAGAGAAGAAGC<br>AAGAGGAGAAAACGGATGTCTTCAGGAACTGAGGAGTGTGGG<br>GAGGACAGGCTCCCCAAGTCTGACTCAGAAGATGGTCCCAGA<br>GCAATGAATCATCTCAGCCTCACCCGTGGCCTCAGCAGGACT<br>TCTATGAAGCCACGTTCCAGCCGCGGGAGCATTTTCACCTTT<br>CGCAGGCGAGACCTGGGTTCTGAAGCAGATTTTGCAGATGAT<br>GAAAACAGCACAGCGGGGGGAGAGCGAGAGCCACCACGCATC<br>ACTGCTGGTGCCCTGGCCCCTGCGCCGGACCAGTGCCCAGGG<br>ACAGCCCAGTCCCGGAACCTCGGCTCCTGGCCACGCCCTCCA<br>TGGCAAAAAGAACAGCACTGTGGACTGCAATGGGGTGGTCTC<br>ATTACTGGGGGCAGGCGACCCAGAGGCCACATCCCCAGGAAG<br>CCACCTCCTCCGCCCTGTGATGCTAGAGCACCCGCCAGACAC<br>GACCACGCCATCGGAGGAGCCAGGCGGGCCCCAGATGCTGAC<br>CTCCCAGGCTCCGTGTGTAGATGGCTTCGAGGAGCCAGGAGC<br>ACGGCAGCGGGCCCTCAGCGCAGTCAGCGTCCTCACCAGCGC<br>ACTGGAAGAGTTAGAGGAGTCTCGCCATAAGTGTCCACCATG<br>CTGGAACCGTCTCGCCCAGCGCTACCTGATCTGGGAGTGCTG<br>CCCGCTGTGGATGTCCATCAAGCAGGGAGTGAAGTTGGTGGT<br>CATGGACCCGTTTACTGACCTCACCATCACTATGTGCATCGT<br>ACTCAACACACTCTTCATGGCGCTGGAGCACTACAACATGAC<br>AAGTGAATTCGAGGAGATGCTGCAGGTCGGAAACCTGGTCTT<br>CACAGGGATTTTCACAGCAGAGATGACCTTCAAGATCATTGC<br>CCTCGACCCCTACTACTACTTCCAACAGGGCTGGAACATCTT<br>CGACAGCATCATCGTCATCCTTAGCCTCATGGAGCTGGGCCT<br>GTCCCGCATGAGCAACTTGTCGGTGCTGCGCTCCTTCCGCCT<br>GCTGCGGGTCTTCAAGCTGGCCAAATCATGGCCCACCCTGAA<br>CACACTCATCAAGATCATCGGGAACTCAGTGGGGGCACTGGG<br>GAACCTGACACTGGTGCTAGCCATCATCGTGTTCATCTTTGC<br>TGTGGTGGGCATGCAGCTCTTTGGCAAGAACTACTCGGAGCT<br>GAGGGACAGCGACTCAGGCCTGCTGCCTCGCTGGCACATGAT<br>GGACTTCTTTCATGCCTTCCTCATCATCTTCCGCATCCTCTG

		TGGAGAGTGGATCGAGACCATGTGGGACTGCATGGAGGTGTC<br>GGGGCAGTCATTATGCCTGCTGGTCTTCTTGCTTGTTATGGT<br>CATTGGCAACCTTGTGGTCCTGAATCTCTTCCTGGCCTTGCT<br>GCTCAGCTCCTTCAGTGCAGACAACCTCACAGCCCCTGATGA<br>GGACAGAGAAGATGAACAACCTCCAAGCTGGCCCTGGCCCGC<br>ATCCAGAAGGGGCCTGCGCTTTGTCAAGCGGACCACCTGGGA<br>TTTCTGCTGTGGTCTCCTGCGGCAGCGGCCTCAGAAGCCCGC<br>AGCCCTTGCCGCCCAGGGCCAGCTGCCCAGCTGCATTGCCAC<br>CCCCTACTCCCCGCCACCCCCAGAGACGGAGAAGGTGCCTCC<br>CACCCGCAAGGAAACACGGTTTGAGGAAGGCGAGCAACCAGG<br>CCAGGGCACCCCCGGGGATCCAGAGCCCGTGTGTGTGCCCAT<br>CGCTGTGGCCGAGTCAGACACAGATGACCAAGAAGAAGATGA<br>GGAGAACAGCCTGGGCACGGAGGAGGAGTCCAGCAAGCAGGA<br>ATCCCAGCCTGTGTCCGGTGGCCCAGAGGCCCCTCCGGATTC<br>CAGGACCTGGAGCCAGGTGTCAGCGACTGCCTCCTCTGAGGC<br>CGAGGCCAGTGCATCTCAGGCCGACTGGCGGCAGCAGTGGAA<br>AGCGGAACCCCAGGCCCCAGGGTGCGGTGAGACCCCAGAGGA<br>CAGTTGCTCCGAGGGCAGCACAGCAGACATGACCAACACCGC<br>TGAGCTCCTGGAGCAGATCCCTGACCTCGGCCAGGATGTCAA<br>GGACCCAGAGGACTGCTTCACTGAAGGCTGTGTCCGGCGCTG<br>TCCCTGCTGTGCGGTGGACACCACACAGGCCCCAGGGAAGGT<br>CTGGTGGCGGTTGCGCAAGACCTGCTACCACATCGTGGAGCA<br>CAGCTGGTTCGAGACATTCATCATCTTCATGATCCTACTCAG<br>CAGTGGAGCGCTGGCCTTCGAGGACATCTACCTAGAGGAGCG<br>GAAGACCATCAAGGTTCTGCTTGAGTATGCCGACAAGATGTT<br>CACATATGTCTTCGTGCTGGAGATGCTGCTCAAGTGGGTGGC<br>CTACGGCTTCAAGAAGTACTTCACCAATGCCTGGTGCTGGCT<br>CGACTTCCTCATCGTAGACGTCTCTCTGGTCAGCCTGGTGGC<br>CAACACCCTGGGCTTTGCCGAGATGGGCCCCATCAAGTCACT<br>GCGGACGCTGCGTGCACTCCGTCCTCTGAGAGCTCTGTCACG<br>ATTTGAGGGCATGAGGGTGGTGGTCAATGCCCTGGTGGGCGC<br>CATCCCGTCCATCATGAACGTCCTCCTCGTCTGCCTCATCTT<br>CTGGCTCATCTTCAGCATCATGGGCGTGAACCTCTTTGCGGG<br>GAAGTTTGGGAGGTGCATCAACCAGACAGAGGGAGACTTGCC<br>TTTGAACTACACCATCGTGAACAACAAGAGCCAGTGTGAGTC<br>CTTGAACTTGACCGGAGAATTGTACTGGACCAAGGTGAAAGT<br>CAACTTTGACAACGTGGGGGCCGGGTACCTGGCCCTTCTGCA<br>GGTGGCAACATTTAAAGGCTGGATGGACATTATGTATGCAGC<br>TGTGGACTCCAGGGGGTATGAAGAGCAGCCTCAGTGGGAATA<br>CAACCTCTACATGTACATCTATTTTGTCATTTTCATCATCTT<br>TGGGTCTTTCTTCACCCTGAACCTCTTTATTGGTGTCATCAT<br>TGACAACTTCAACCAACAGAAGAAAAAGTTAGGGGGCCAGGA<br>CATCTTCATGACAGAGGAGCAGAAGAAGTACTACAATGCCAT<br>GAAGAAGCTGGGCTCCAAGAAGCCCCAGAAGCCCATCCCACG

		GCCCCTGAACAAGTACCAGGGCTTCATATTCGACATTGTGAC<br>CAAGCAGGCCTTTGACGTCACCATCATGTTTCTGATCTGCTT<br>GAATATGGTGACCATGATGGTGGAGACAGATGACCAAAGTCC<br>TGAGAAAATCAACATCTTGGCCAAGATCAACCTGCTCTTTGT<br>GGCCATCTTCACAGGCGAGTGTATTGTCAAGCTGGCTGCCCT<br>GCGCCACTACTACTTCACCAACAGCTGGAATATCTTCGACTT<br>CGTGGTTGTCATCCTCTCCATCGTGGGCACTGTGCTCTCGGA<br>CATCATCCAGAAGTACTTCTTCTCCCCGACGCTCTTCCGAGT<br>CATCCGCCTGGCCCGAATAGGCCGCATCCTCAGACTGATCCG<br>AGGGGCCAAGGGGATCCGCACGCTGCTCTTTGCCCTCATGAT<br>GTCCCTGCCTGCCCTCTTCAACATCGGGCTGCTG
Insert-6	6 kb	ATGGCAAACTTCCTATTACCTCGGGGCACCAGCAGCTTCCGC<br>AGGTTCACACGGGAGTCCCTGGCAGCCATCGAGAAGCGCATG<br>GCGGAGAAGCAAGCCCGCGGCTCAACCACCTTGCAGGAGAGC<br>CGAGAGGGGCTGCCCGAGGAGGAGGCTCCCCGGCCCCAGCTG<br>GACCTGCAGGCCTCCAAAAAGCTGCCAGATCTCTATGGCAAT<br>CCACCCCAAGAGCTCATCGGAGAGCCCCTGGAGGACCTGGAC<br>CCCTTCTATAGCACCCAAAAGACTTTCATCGTACTGAATAAA<br>GGCAAGACCATCTTCCGGTTCAGTGCCACCAACGCCTTGTAT<br>GTCCTCAGTCCCTTCCACCCCATCCGGAGAGCGGCTGTGAAG<br>ATTCTGGTTCACTCGCTCTTCAACATGCTCATCATGTGCACC<br>ATCCTCACCAACTGCGTGTTCATGGCCCAGCACGACCCTCCA<br>CCCTGGACCAAGTATGTCGAGTACACCTTCACCGCCATTTAC<br>ACCTTTGAGTCTCTGGTCAAGATTCTGGCTCGAGGCTTCTGC<br>CTGCACGCGTTCACTTTCCTTCGGGACCCATGGAACTGGCTG<br>GACTTTAGTGTGATTATCATGGCATACACAACTGAATTTGTG<br>GACCTGGGCAATGTCTCAGCCTTACGCACCTTCCGAGTCCTC<br>CGGGCCCTGAAAACTATATCAGTCATTTCAGGGCTGAAGACC<br>ATCGTGGGGGCCCTGATCCAGTCTGTGAAGAAGCTGGCTGAT<br>GTGATGGTCCTCACAGTCTTCTGCCTCAGCGTCTTTGCCCTC<br>ATCGGCCTGCAGCTCTTCATGGGCAACCTAAGGCACAAGTGC<br>GTGCGCAACTTCACAGCGCTCAACGGCACCAACGGCTCCGTG<br>GAGGCCGACGGCTTGGTCTGGGAATCCCTGGACCTTTACCTC<br>AGTGATCCAGAAAATTACCTGCTCAAGAACGGCACCTCTGAT<br>GTGTTACTGTGTGGGAACAGCTCTGACGCTGGGACATGTCCG<br>GAGGGCTACCGGTGCCTAAAGGCAGGCGAGAACCCCGACCAC<br>GGCTACACCAGCTTCGATTCCTTTGCCTGGGCCTTTCTTGCA<br>CTCTTCCGCCTGATGACGCAGGACTGCTGGGAGCGCCTCTAT<br>CAGCAGACCCTCAGGTCCGCAGGGAAGATCTACATGATCTTC<br>TTCATGCTTGTCATCTTCCTGGGGTCCTTCTACCTGGTGAAC<br>CTGATCCTGGCCGTGGTCGCAATGGCCTATGAGGAGCAAAAC<br>CAAGCCACCATCGCTGAGACCGAGGAGAAGGAAAAGCGCTTC<br>CAGGAGGCCATGGAAATGCTCAAGAAAGAACACGAGGCCCTC<br>ACCATCAGGGGTGTGGATACCGTGTCCCGTAGCTCCTTGGAG

		ATGTCCCCTTTGGCCCCAGTAAACAGCCATGAGAGAAGAAGC<br>AAGAGGAGAAAACGGATGTCTTCAGGAACTGAGGAGTGTGGG<br>GAGGACAGGCTCCCCAAGTCTGACTCAGAAGATGGTCCCAGA<br>GCAATGAATCATCTCAGCCTCACCCGTGGCCTCAGCAGGACT<br>TCTATGAAGCCACGTTCCAGCCGCGGGAGCATTTTCACCTTT<br>CGCAGGCGAGACCTGGGTTCTGAAGCAGATTTTGCAGATGAT<br>GAAAACAGCACAGCGGGGGGAGAGCGAGAGCCACCACGCATC<br>ACTGCTGGTGCCCTGGCCCCTGCGCCGGACCAGTGCCCAGGG<br>ACAGCCCAGTCCCGGAACCTCGGCTCCTGGCCACGCCCTCCA<br>TGGCAAAAAGAACAGCACTGTGGACTGCAATGGGGTGGTCTC<br>ATTACTGGGGGCAGGCGACCCAGAGGCCACATCCCCAGGAAG<br>CCACCTCCTCCGCCCTGTGATGCTAGAGCACCCGCCAGACAC<br>GACCACGCCATCGGAGGAGCCAGGCGGGCCCCAGATGCTGAC<br>CTCCCAGGCTCCGTGTGTAGATGGCTTCGAGGAGCCAGGAGC<br>ACGGCAGCGGGCCCTCAGCGCAGTCAGCGTCCTCACCAGCGC<br>ACTGGAAGAGTTAGAGGAGTCTCGCCATAAGTGTCCACCATG<br>CTGGAACCGTCTCGCCCAGCGCTACCTGATCTGGGAGTGCTG<br>CCCGCTGTGGATGTCCATCAAGCAGGGAGTGAAGTTGGTGGT<br>CATGGACCCGTTTACTGACCTCACCATCACTATGTGCATCGT<br>ACTCAACACACTCTTCATGGCGCTGGAGCACTACAACATGAC<br>AAGTGAATTCGAGGAGATGCTGCAGGTCGGAAACCTGGTCTT<br>CACAGGGATTTTCACAGCAGAGATGACCTTCAAGATCATTGC<br>CCTCGACCCCTACTACTACTTCCAACAGGGCTGGAACATCTT<br>CGACAGCATCATCGTCATCCTTAGCCTCATGGAGCTGGGCCT<br>GTCCCGCATGAGCAACTTGTCGGTGCTGCGCTCCTTCCGCCT<br>GCTGCGGGTCTTCAAGCTGGCCAAATCATGGCCCACCCTGAA<br>CACACTCATCAAGATCATCGGGAACTCAGTGGGGGCACTGGG<br>GAACCTGACACTGGTGCTAGCCATCATCGTGTTCATCTTTGC<br>TGTGGTGGGCATGCAGCTCTTTGGCAAGAACTACTCGGAGCT<br>GAGGGACAGCGACTCAGGCCTGCTGCCTCGCTGGCACATGAT<br>GGACTTCTTTCATGCCTTCCTCATCATCTTCCGCATCCTCTG<br>TGGAGAGTGGATCGAGACCATGTGGGACTGCATGGAGGTGTC<br>GGGGCAGTCATTATGCCTGCTGGTCTTCTTGCTTGTTATGGT<br>CATTGGCAACCTTGTGGTCCTGAATCTCTTCCTGGCCTTGCT<br>GCTCAGCTCCTTCAGTGCAGACAACCTCACAGCCCCTGATGA<br>GGACAGAGAAGATGAACAACCTCCAAGCTGGCCCTGGCCCGC<br>ATCCAGAAGGGGCCTGCGCTTTGTCAAGCGGACCACCTGGGA<br>TTTCTGCTGTGGTCTCCTGCGGCAGCGGCCTCAGAAGCCCGC<br>AGCCCTTGCCGCCCAGGGCCAGCTGCCCAGCTGCATTGCCAC<br>CCCCTACTCCCCGCCACCCCCAGAGACGGAGAAGGTGCCTCC<br>CACCCGCAAGGAAACACGGTTTGAGGAAGGCGAGCAACCAGG<br>CCAGGGCACCCCCGGGGATCCAGAGCCCGTGTGTGTGCCCAT<br>CGCTGTGGCCGAGTCAGACACAGATGACCAAGAAGAAGATGA<br>GGAGAACAGCCTGGGCACGGAGGAGGAGTCCAGCAAGCAGGA

		ATCCCAGCCTGTGTCCGGTGGCCCAGAGGCCCCTCCGGATTC<br>CAGGACCTGGAGCCAGGTGTCAGCGACTGCCTCCTCTGAGGC<br>CGAGGCCAGTGCATCTCAGGCCGACTGGCGGCAGCAGTGGAA<br>AGCGGAACCCCAGGCCCCAGGGTGCGGTGAGACCCCAGAGGA<br>CAGTTGCTCCGAGGGCAGCACAGCAGACATGACCAACACCGC<br>TGAGCTCCTGGAGCAGATCCCTGACCTCGGCCAGGATGTCAA<br>GGACCCAGAGGACTGCTTCACTGAAGGCTGTGTCCGGCGCTG<br>TCCCTGCTGTGCGGTGGACACCACACAGGCCCCAGGGAAGGT<br>CTGGTGGCGGTTGCGCAAGACCTGCTACCACATCGTGGAGCA<br>CAGCTGGTTCGAGACATTCATCATCTTCATGATCCTACTCAG<br>CAGTGGAGCGCTGGCCTTCGAGGACATCTACCTAGAGGAGCG<br>GAAGACCATCAAGGTTCTGCTTGAGTATGCCGACAAGATGTT<br>CACATATGTCTTCGTGCTGGAGATGCTGCTCAAGTGGGTGGC<br>CTACGGCTTCAAGAAGTACTTCACCAATGCCTGGTGCTGGCT<br>CGACTTCCTCATCGTAGACGTCTCTCTGGTCAGCCTGGTGGC<br>CAACACCCTGGGCTTTGCCGAGATGGGCCCCATCAAGTCACT<br>GCGGACGCTGCGTGCACTCCGTCCTCTGAGAGCTCTGTCACG<br>ATTTGAGGGCATGAGGGTGGTGGTCAATGCCCTGGTGGGCGC<br>CATCCCGTCCATCATGAACGTCCTCCTCGTCTGCCTCATCTT<br>CTGGCTCATCTTCAGCATCATGGGCGTGAACCTCTTTGCGGG<br>GAAGTTTGGGAGGTGCATCAACCAGACAGAGGGAGACTTGCC<br>TTTGAACTACACCATCGTGAACAACAAGAGCCAGTGTGAGTC<br>CTTGAACTTGACCGGAGAATTGTACTGGACCAAGGTGAAAGT<br>CAACTTTGACAACGTGGGGGCCGGGTACCTGGCCCTTCTGCA<br>GGTGGCAACATTTAAAGGCTGGATGGACATTATGTATGCAGC<br>TGTGGACTCCAGGGGGTATGAAGAGCAGCCTCAGTGGGAATA<br>CAACCTCTACATGTACATCTATTTTGTCATTTTCATCATCTT<br>TGGGTCTTTCTTCACCCTGAACCTCTTTATTGGTGTCATCAT<br>TGACAACTTCAACCAACAGAAGAAAAAGTTAGGGGGCCAGGA<br>CATCTTCATGACAGAGGAGCAGAAGAAGTACTACAATGCCAT<br>GAAGAAGCTGGGCTCCAAGAAGCCCCAGAAGCCCATCCCACG<br>GCCCCTGAACAAGTACCAGGGCTTCATATTCGACATTGTGAC<br>CAAGCAGGCCTTTGACGTCACCATCATGTTTCTGATCTGCTT<br>GAATATGGTGACCATGATGGTGGAGACAGATGACCAAAGTCC<br>TGAGAAAATCAACATCTTGGCCAAGATCAACCTGCTCTTTGT<br>GGCCATCTTCACAGGCGAGTGTATTGTCAAGCTGGCTGCCCT<br>GCGCCACTACTACTTCACCAACAGCTGGAATATCTTCGACTT<br>CGTGGTTGTCATCCTCTCCATCGTGGGCACTGTGCTCTCGGA<br>CATCATCCAGAAGTACTTCTTCTCCCCGACGCTCTTCCGAGT<br>CATCCGCCTGGCCCGAATAGGCCGCATCCTCAGACTGATCCG<br>AGGGGCCAAGGGGATCCGCACGCTGCTCTTTGCCCTCATGAT<br>GTCCCTGCCTGCCCTCTTCAACATCGGGCTGCTGCTCTTCCT<br>CGTCATGTTCATCTACTCCATCTTTGGCATGGCCAACTTCGC<br>TTATGTCAAGTGGGAGGCTGGCATCGACGACATGTTCAACTT

		CCAGACCTTCGCCAACAGCATGCTGTGCCTCTTCCAGATCAC<br>CACGTCGGCCGGCTGGGATGGCCTCCTCAGCCCCATCCTCAA<br>CACTGGGCCGCCCTACTGCGACCCCACTCTGCCCAACAGCAA<br>TGGCTCTCGGGGGGACTGCGGGAGCCCAGCCGTGGGCATCCT<br>CTTCTTCACCACCTACATCATCATCTCCTTCCTCATCGTGGT<br>CAACATGTACATTGCCATCATCCTGGAGAACTTCAGCGTGGC<br>CACGGAGGAGAGCACCGAGCCCCTGAGTGAGGACGACTTCGA<br>TATGTTCTATGAGATCTGGGAGAAATTTGACCCAGAGGCCAC<br>TCAGTTTATTGAGTATTCGGTCCTGTCTGACTTTGCCGATGC<br>CCTGTCTGAGCCACTCCGTATCGCCAAGCCCAACCAGATAAG<br>CCTCATCAACATGGACCTGCCCATGGTGAGTGGGGACCGCAT<br>CCATTGCATGGACATTCTCTTTGCCTTCACCAAAAGGGTCCT<br>GGGGGAGTCTGGGGAGATGGACGCCCTGAAGATCCAGATGGA<br>GGAGAAGTTCATGGCAGCCAACCCATCCAAGATCTCCTACGA<br>GCCCATCACCACCACACTCCGGCGCAAGCACGAAGAGGTGTC<br>GGCCATGGTTATCCAGAGAGCCTTCCGCAGGCACCTGCTGCA<br>ACGCTCTTTGAAGCATGCCTCCTTCCTCTTCCGTCAGCAGGC<br>GGGCAGCGGCCTCTCCGAAGAGGATGCCCCTGAGCGAGAGGG<br>CCTCATCGCCTACGTGATGAGTGAGAACTTCTCCCGACCCCT<br>TGGCCCACCCTCCAGCTCCTCCATCTCCTCCACTTCCTTCCC<br>ACCCTCCTATGACAGTGTCACTAGAGCCACCAGCGATAACCT<br>CCAGGTGCGGGGGTCTGACTACAGC
Insert-7	two 1 kb	ATGGCAAACTTCCTATTACCTCGGGGCACCAGCAGCTTCCGC<br>AGGTTCACACGGGAGTCCCTGGCAGCCATCGAGAAGCGCATG<br>GCGGAGAAGCAAGCCCGCGGCTCAACCACCTTGCAGGAGAGC<br>CGAGAGGGGCTGCCCGAGGAGGAGGCTCCCCGGCCCCAGCTG<br>GACCTGCAGGCCTCCAAAAAGCTGCCAGATCTCTATGGCAAT<br>CCACCCCAAGAGCTCATCGGAGAGCCCCTGGAGGACCTGGAC<br>CCCTTCTATAGCACCCAAAAGACTTTCATCGTACTGAATAAA<br>GGCAAGACCATCTTCCGGTTCAGTGCCACCAACGCCTTGTAT<br>GTCCTCAGTCCCTTCCACCCCATCCGGAGAGCGGCTGTGAAG<br>ATTCTGGTTCACTCGCTCTTCAACATGCTCATCATGTGCACC<br>ATCCTCACCAACTGCGTGTTCATGGCCCAGCACGACCCTCCA<br>CCCTGGACCAAGTATGTCGAGTACACCTTCACCGCCATTTAC<br>ACCTTTGAGTCTCTGGTCAAGATTCTGGCTCGAGGCTTCTGC<br>CTGCACGCGTTCACTTTCCTTCGGGACCCATGGAACTGGCTG<br>GACTTTAGTGTGATTATCATGGCATACACAACTGAATTTGTG<br>GACCTGGGCAATGTCTCAGCCTTACGCACCTTCCGAGTCCTC<br>CGGGCCCTGAAAACTATATCAGTCATTTCAGGGCTGAAGACC<br>ATCGTGGGGGCCCTGATCCAGTCTGTGAAGAAGCTGGCTGAT<br>GTGATGGTCCTCACAGTCTTCTGCCTCAGCGTCTTTGCCCTC<br>ATCGGCCTGCAGCTCTTCATGGGCAACCTAAGGCACAAGTGC<br>GTGCGCAACTTCACAGCGCTCAACGGCACCAACGGCTCCGTG<br>GAGGCCGACGGCTTGGTCTGGGAATCCCTGGACCTTTACCTC

		AGTGATCCAGAAAATTACCTGCTCAAGAACGGCACCTCTGAT<br>GTGTTACTGTGTGGGAACAGCTCTGACGCTGGGA<br>ATGGCCAGCTCATCTCTGCCCAACCTGGTCCCCCGGGTCCC<br>CACTGCCTGCGCCCCTTCACCCCAGAGTCCCTGGCAGCCATA<br>GAGCAGCGGGCGGTGGAGGAGGAGGCCCGGCTGCAGCGGAAC<br>AAGCAGATGGAGATTGAGGAGCCTGAGCGGAAGCCACGCAGT<br>GACCTGGAAGCTGGCAAGAACCTCCCACTCATCTATGGGGAC<br>CCCCCACCCGAAGTCATTGGCATCCCCCTGGAGGACCTGGAT<br>CCTTACTACAGTGACAAGAAGACCTTCATTGTGCTCAACAAA<br>GGAAAGGCCATCTTCCGATTCTCTGCCACGCCTGCCCTCTAC<br>CTGCTGAGCCCCTTCAGCATCGTCAGGAGGGTGGCTATCAAG<br>GTGCTCATTCACGCGCTGTTCAGCATGTTTATCATGATCACC<br>ATCCTGACCAACTGTGTGTTCATGACCATGAGCAATCCGCCT<br>TCTTGGTCCAAACACGTGGAGTACACCTTCACGGGGATCTAT<br>ACCTTTGAGTCCCTCATTAAGATGCTGGCCCGAGGCTTTTGC<br>ATTGATGACTTCACATTCCTCCGAGACCCCTGGAACTGGCTG<br>GACTTCAGTGTCATCACAATGGCGTATGTGACAGAGTTTGTG<br>GACTTGGGCAACATCTCAGCCCTGAGGACCTTCCGTGTGCTG<br>CGGGCCCTGAAGACCATCACGGTTATCCCAGGGCTGAAGACA<br>ATTGTGGGAGCCCTGATCCAGTCTGTGAAAAAGCTGTCGGAT<br>GTGATGATCCTCACTGTCTTCTGCCTGAGTGTCTTTGCCCTG<br>GTGGGGCTGCAGCTTTTCATGGGAAACCTGCGTCAGAAGTGC<br>GTGCGTTGGCCCCCGCCCATGAATGACACCAACACCACGTGG<br>TATGGCAATGACACTTGGTACAGCAATGACACTTGGTACGGC<br>AATGACACTTGGTACATCAATGACACTTGGAACAGCCAGGAG<br>AGCTGGGCCGGCAACTCTACCTTTGACTGGGAGG
Insert-8	two 2 kb	ATGGCAAACTTCCTATTACCTCGGGGCACCAGCAGCTTCCGC<br>AGGTTCACACGGGAGTCCCTGGCAGCCATCGAGAAGCGCATG<br>GCGGAGAAGCAAGCCCGCGGCTCAACCACCTTGCAGGAGAGC<br>CGAGAGGGGCTGCCCGAGGAGGAGGCTCCCCGGCCCCAGCTG<br>GACCTGCAGGCCTCCAAAAAGCTGCCAGATCTCTATGGCAAT<br>CCACCCCAAGAGCTCATCGGAGAGCCCCTGGAGGACCTGGAC<br>CCCTTCTATAGCACCCAAAAGACTTTCATCGTACTGAATAAA<br>GGCAAGACCATCTTCCGGTTCAGTGCCACCAACGCCTTGTAT<br>GTCCTCAGTCCCTTCCACCCCATCCGGAGAGCGGCTGTGAAG<br>ATTCTGGTTCACTCGCTCTTCAACATGCTCATCATGTGCACC<br>ATCCTCACCAACTGCGTGTTCATGGCCCAGCACGACCCTCCA<br>CCCTGGACCAAGTATGTCGAGTACACCTTCACCGCCATTTAC<br>ACCTTTGAGTCTCTGGTCAAGATTCTGGCTCGAGGCTTCTGC<br>CTGCACGCGTTCACTTTCCTTCGGGACCCATGGAACTGGCTG<br>GACTTTAGTGTGATTATCATGGCATACACAACTGAATTTGTG<br>GACCTGGGCAATGTCTCAGCCTTACGCACCTTCCGAGTCCTC<br>CGGGCCCTGAAAACTATATCAGTCATTTCAGGGCTGAAGACC

		ATCGTGGGGGCCCTGATCCAGTCTGTGAAGAAGCTGGCTGAT<br>GTGATGGTCCTCACAGTCTTCTGCCTCAGCGTCTTTGCCCTC<br>ATCGGCCTGCAGCTCTTCATGGGCAACCTAAGGCACAAGTGC<br>GTGCGCAACTTCACAGCGCTCAACGGCACCAACGGCTCCGTG<br>GAGGCCGACGGCTTGGTCTGGGAATCCCTGGACCTTTACCTC<br>AGTGATCCAGAAAATTACCTGCTCAAGAACGGCACCTCTGAT<br>GTGTTACTGTGTGGGAACAGCTCTGACGCTGGGACATGTCCG<br>GAGGGCTACCGGTGCCTAAAGGCAGGCGAGAACCCCGACCAC<br>GGCTACACCAGCTTCGATTCCTTTGCCTGGGCCTTTCTTGCA<br>CTCTTCCGCCTGATGACGCAGGACTGCTGGGAGCGCCTCTAT<br>CAGCAGACCCTCAGGTCCGCAGGGAAGATCTACATGATCTTC<br>TTCATGCTTGTCATCTTCCTGGGGTCCTTCTACCTGGTGAAC<br>CTGATCCTGGCCGTGGTCGCAATGGCCTATGAGGAGCAAAAC<br>CAAGCCACCATCGCTGAGACCGAGGAGAAGGAAAAGCGCTTC<br>CAGGAGGCCATGGAAATGCTCAAGAAAGAACACGAGGCCCTC<br>ACCATCAGGGGTGTGGATACCGTGTCCCGTAGCTCCTTGGAG<br>ATGTCCCCTTTGGCCCCAGTAAACAGCCATGAGAGAAGAAGC<br>AAGAGGAGAAAACGGATGTCTTCAGGAACTGAGGAGTGTGGG<br>GAGGACAGGCTCCCCAAGTCTGACTCAGAAGATGGTCCCAGA<br>GCAATGAATCATCTCAGCCTCACCCGTGGCCTCAGCAGGACT<br>TCTATGAAGCCACGTTCCAGCCGCGGGAGCATTTTCACCTTT<br>CGCAGGCGAGACCTGGGTTCTGAAGCAGATTTTGCAGATGAT<br>GAAAACAGCACAGCGGGGGAGAGCGAGAGCCACCACGCATC<br>ACTGCTGGTGCCCTGGCCCCTGCGCCGGACCAGTGCCCAGGG<br>ACAGCCCAGTCCCGGAACCTCGGCTCCTGGCCACGCCCTCCA<br>TGGCAAAAAGAACAGCACTGTGGACTGCAATGGGGTGGTCTC<br>ATTACTGGGGGCAGGCGACCCAGAGGCCACATCCCCAGGAAG<br>CCACCTCCTCCGCCCTGTGATGCTAGAGCACCCGCCAGACAC<br>GACCACGCCATCGGAGGAGCCAGGCGGGCCCCAGATGCTGAC<br>CTCCCAGGCTCCGTGTGTAGATGGCTTCGAGGAGCCAGGAGC<br>ACGGCAGCGGGCCCTCAGCGCAGTCA<br>ATGGCCAGCTCATCTCTGCCCAACCTGGTCCCCCCGGGTCCC<br>CACTGCCTGCGCCCCTTCACCCCAGAGTCCCTGGCAGCCATA<br>GAGCAGCGGGCGGTGGAGGAGGAGGCCCGGCTGCAGCGGAAC<br>AAGCAGATGGAGATTGAGGAGCCTGAGCGGAAGCCACGCAGT<br>GACCTGGAAGCTGGCAAGAACCTCCCACTCATCTATGGGGAC<br>CCCCCACCCGAAGTCATTGGCATCCCCCTGGAGGACCTGGAT<br>CCTTACTACAGTGACAAGAAGACCTTCATTGTGCTCAACAAA<br>GGAAAGGCCATCTTCCGATTCTCTGCCACGCCTGCCCTCTAC<br>CTGCTGAGCCCCTTCAGCATCGTCAGGAGGGTGGCTATCAAG<br>GTGCTCATTCACGCGCTGTTCAGCATGTTTATCATGATCACC<br>ATCCTGACCAACTGTGTGTTCATGACCATGAGCAATCCGCCT<br>TCTTGGTCCAAACACGTGGAGTACACCTTCACGGGGATCTAT<br>ACCTTTGAGTCCCTCATTAAGATGCTGGCCCGAGGCTTTTGC

		ATTGATGACTTCACATTCCTCCGAGACCCCTGGAACTGGCTG<br>GACTTCAGTGTCATCACAATGGCGTATGTGACAGAGTTTGTG<br>GACTTGGGCAACATCTCAGCCCTGAGGACCTTCCGTGTGCTG<br>CGGGCCCTGAAGACCATCACGGTTATCCCAGGGCTGAAGACA<br>ATTGTGGGAGCCCTGATCCAGTCTGTGAAAAAGCTGTCGGAT<br>GTGATGATCCTCACTGTCTTCTGCCTGAGTGTCTTTGCCCTG<br>GTGGGGCTGCAGCTTTTCATGGGAAACCTGCGTCAGAAGTGC<br>GTGCGTTGGCCCCCGCCCATGAATGACACCAACACCACGTGG<br>TATGGCAATGACACTTGGTACAGCAATGACACTTGGTACGGC<br>AATGACACTTGGTACATCAATGACACTTGGAACAGCCAGGAG<br>AGCTGGGCCGGCAACTCTACCTTTGACTGGGAGGCCTACATC<br>AATGACGAAGGGAACTTCTATTTCTTGGAGGGCTCCAATGAT<br>GCTCTGCTCTGTGGGAATAGCAGTGATGCTGGGCACTGCCCT<br>GAGGGCTACGAATGCATAAAGGCTGGGCGGAACCCCAACTAT<br>GGCTACACCAGCTATGACACCTTCAGCTGGGCTTTCCTGGCT<br>CTCTTCCGGCTCATGACGCAGGACTACTGGGAGAACCTTTTC<br>CAGCTGACCCTACGAGCTGCTGGCAAGACCTACATGATCTTC<br>TTCGTGGTCATCATCTTCCTGGGCTCCTTCTACCTCATCAAT<br>CTGATCCTGGCCGTGGTGGCCATGGCGTACGCTGAGCAGAAT<br>GAGGCTACCCTGGCCGAAGACCAGGAGAAAGAGGAGGAGTTC<br>CAACAGATGCTTGAGAAATACAAAAAACATCAGGAGGAACTG<br>GAAAAGGCTAAGGCTGCCCAGGCTCTGGAAAGTGGAGAGGAG<br>GCAGATGGGGACCCAACCCACAACAAAGACTGCAATGGGAGC<br>CTGGATGCATCCGGGGAGAAGGGGCCCCCAAGGCCAAGCTGC<br>AGCGCAGACAGTGCCATCTCAGATGCTATGGAGGAGCTGGAA<br>GAGGCCCATCAGAAGTGCCCACCGTGGTGGTACAAGTGTGCA<br>CACAAAGTCCTCATCTGGAACTGCTGTGCCCCGTGGGTGAAG<br>TTCAAACATATAATCTACCTGATCGTCATGGACCCCTTTGTG<br>GACCTGGGTATCACCATCTGCATTGTGCTCAACACCCTCTTC<br>ATGGCCATGGAGCACTACCCCATGACCGAGCACTTTGACAAC<br>GTGCTCTCCGTGGGCAACTTGGTCTTCACAGGCATCTTCACT<br>GCGGAGATGGTGTTGAAGCTGATTGCCATGGACCCCTACGAG<br>TATTTCCAACAGGGCTGGAACATCTTTGACAGTTTCATCGTC<br>ACCCTCAGCCTGGTGGAGCTGGGCCTGGCCAACGTACAGGGG<br>CTGTCAGTGCTCCGTTCCTTCCGC
Insert-9	two 3 kb	ATGGCAAACTTCCTATTACCTCGGGGCACCAGCAGCTTCCGC<br>AGGTTCACACGGGAGTCCCTGGCAGCCATCGAGAAGCGCATG<br>GCGGAGAAGCAAGCCCGCGGCTCAACCACCTTGCAGGAGAGC<br>CGAGAGGGGCTGCCCGAGGAGGAGGCTCCCCGGCCCCAGCTG<br>GACCTGCAGGCCTCCAAAAAGCTGCCAGATCTCTATGGCAAT<br>CCACCCCAAGAGCTCATCGGAGAGCCCCTGGAGGACCTGGAC<br>CCCTTCTATAGCACCCAAAAGACTTTCATCGTACTGAATAAA<br>GGCAAGACCATCTTCCGGTTCAGTGCCACCAACGCCTTGTAT<br>GTCCTCAGTCCCTTCCACCCCATCCGGAGAGCGGCTGTGAAG

		ATTCTGGTTCACTCGCTCTTCAACATGCTCATCATGTGCACC<br>ATCCTCACCAACTGCGTGTTCATGGCCCAGCACGACCCTCCA<br>CCCTGGACCAAGTATGTCGAGTACACCTTCACCGCCATTTAC<br>ACCTTTGAGTCTCTGGTCAAGATTCTGGCTCGAGGCTTCTGC<br>CTGCACGCGTTCACTTTCCTTCGGGACCCATGGAACTGGCTG<br>GACTTTAGTGTGATTATCATGGCATACACAACTGAATTTGTG<br>GACCTGGGCAATGTCTCAGCCTTACGCACCTTCCGAGTCCTC<br>CGGGCCCTGAAAACTATATCAGTCATTTCAGGGCTGAAGACC<br>ATCGTGGGGGCCCTGATCCAGTCTGTGAAGAAGCTGGCTGAT<br>GTGATGGTCCTCACAGTCTTCTGCCTCAGCGTCTTTGCCCTC<br>ATCGGCCTGCAGCTCTTCATGGGCAACCTAAGGCACAAGTGC<br>GTGCGCAACTTCACAGCGCTCAACGGCACCAACGGCTCCGTG<br>GAGGCCGACGGCTTGGTCTGGGAATCCCTGGACCTTTACCTC<br>AGTGATCCAGAAAATTACCTGCTCAAGAACGGCACCTCTGAT<br>GTGTTACTGTGTGGGAACAGCTCTGACGCTGGGACATGTCCG<br>GAGGGCTACCGGTGCCTAAAGGCAGGCGAGAACCCCGACCAC<br>GGCTACACCAGCTTCGATTCCTTTGCCTGGGCCTTTCTTGCA<br>CTCTTCCGCCTGATGACGCAGGACTGCTGGGAGCGCCTCTAT<br>CAGCAGACCCTCAGGTCCGCAGGGAAGATCTACATGATCTTC<br>TTCATGCTTGTCATCTTCCTGGGGTCCTTCTACCTGGTGAAC<br>CTGATCCTGGCCGTGGTCGCAATGGCCTATGAGGAGCAAAAC<br>CAAGCCACCATCGCTGAGACCGAGGAGAAGGAAAAGCGCTTC<br>CAGGAGGCCATGGAAATGCTCAAGAAAGAACACGAGGCCCTC<br>ACCATCAGGGGTGTGGATACCGTGTCCCGTAGCTCCTTGGAG<br>ATGTCCCCTTTGGCCCCAGTAAACAGCCATGAGAGAAGAAGC<br>AAGAGGAGAAAACGGATGTCTTCAGGAACTGAGGAGTGTGGG<br>GAGGACAGGCTCCCCAAGTCTGACTCAGAAGATGGTCCCAGA<br>GCAATGAATCATCTCAGCCTCACCCGTGGCCTCAGCAGGACT<br>TCTATGAAGCCACGTTCCAGCCGCGGGAGCATTTTCACCTTT<br>CGCAGGCGAGACCTGGGTTCTGAAGCAGATTTTGCAGATGAT<br>GAAAACAGCACAGCGGGGGAGAGCGAGAGCCACCACGCATC<br>ACTGCTGGTGCCCTGGCCCCTGCGCCGGACCAGTGCCCAGGG<br>ACAGCCCAGTCCCGGAACCTCGGCTCCTGGCCACGCCCTCCA<br>TGGCAAAAAGAACAGCACTGTGGACTGCAATGGGGTGGTCTC<br>ATTACTGGGGGCAGGCGACCCAGAGGCCACATCCCCAGGAAG<br>CCACCTCCTCCGCCCTGTGATGCTAGAGCACCCGCCAGACAC<br>GACCACGCCATCGGAGGAGCCAGGCGGGCCCCAGATGCTGAC<br>CTCCCAGGCTCCGTGTGTAGATGGCTTCGAGGAGCCAGGAGC<br>ACGGCAGCGGGCCCTCAGCGCAGTCAGCGTCCTCACCAGCGC<br>ACTGGAAGAGTTAGAGGAGTCTCGCCATAAGTGTCCACCATG<br>CTGGAACCGTCTCGCCCAGCGCTACCTGATCTGGGAGTGCTG<br>CCCGCTGTGGATGTCCATCAAGCAGGGAGTGAAGTTGGTGGT<br>CATGGACCCGTTTACTGACCTCACCATCACTATGTGCATCGT<br>ACTCAACACACTCTTCATGGCGCTGGAGCACTACAACATGAC

		AAGTGAATTCGAGGAGATGCTGCAGGTCGGAAACCTGGTCTT<br>CACAGGGATTTTCACAGCAGAGATGACCTTCAAGATCATTGC<br>CCTCGACCCCTACTACTACTTCCAACAGGGCTGGAACATCTT<br>CGACAGCATCATCGTCATCCTTAGCCTCATGGAGCTGGGCCT<br>GTCCCGCATGAGCAACTTGTCGGTGCTGCGCTCCTTCCGCCT<br>GCTGCGGGTCTTCAAGCTGGCCAAATCATGGCCCACCCTGAA<br>CACACTCATCAAGATCATCGGGAACTCAGTGGGGGCACTGGG<br>GAACCTGACACTGGTGCTAGCCATCATCGTGTTCATCTTTGC<br>TGTGGTGGGCATGCAGCTCTTTGGCAAGAACTACTCGGAGCT<br>GAGGGACAGCGACTCAGGCCTGCTGCCTCGCTGGCACATGAT<br>GGACTTCTTTCATGCCTTCCTCATCATCTTCCGCATCCTCTG<br>TGGAGAGTGGATCGAGACCATGTGGGACTGCATGGAGGTGTC<br>GGGGCAGTCATTATGCCTGCTGGTCTTCTTGCTTGTTATGGT<br>CATTGGCAACCTTGTGGTCCTGAATCTCTTCCTGGCCTTGCT<br>GCTCAGCTCCTTCAGTGCAGACAACCTCACAGCCCCTGATGA<br>GGACAGAGAAGATGAACAACCTCCAAGCTGGCCCTGGCCCGC<br>ATCCAGAAGGGGCCTGCGCTTTGTCAAGCGGACCACCTGGGA<br>TTTCTGCTGTGGTCTCCTGCGGCAGCGGCCTCAGAAGCCCGC<br>AGCCCTTGCCGC<br>ATGGCCAGCTCATCTCTGCCCAACCTGGTCCCCCCGGGTCCC<br>CACTGCCTGCGCCCCTTCACCCCAGAGTCCCTGGCAGCCATA<br>GAGCAGCGGGCGGTGGAGGAGGAGGCCCGGCTGCAGCGGAAC<br>AAGCAGATGGAGATTGAGGAGCCTGAGCGGAAGCCACGCAGT<br>GACCTGGAAGCTGGCAAGAACCTCCCACTCATCTATGGGGAC<br>CCCCCACCCGAAGTCATTGGCATCCCCCTGGAGGACCTGGAT<br>CCTTACTACAGTGACAAGAAGACCTTCATTGTGCTCAACAAA<br>GGAAAGGCCATCTTCCGATTCTCTGCCACGCCTGCCCTCTAC<br>CTGCTGAGCCCCTTCAGCATCGTCAGGAGGGTGGCTATCAAG<br>GTGCTCATTCACGCGCTGTTCAGCATGTTTATCATGATCACC<br>ATCCTGACCAACTGTGTGTTCATGACCATGAGCAATCCGCCT<br>TCTTGGTCCAAACACGTGGAGTACACCTTCACGGGGATCTAT<br>ACCTTTGAGTCCCTCATTAAGATGCTGGCCCGAGGCTTTTGC<br>ATTGATGACTTCACATTCCTCCGAGACCCCTGGAACTGGCTG<br>GACTTCAGTGTCATCACAATGGCGTATGTGACAGAGTTTGTG<br>GACTTGGGCAACATCTCAGCCCTGAGGACCTTCCGTGTGCTG<br>CGGGCCCTGAAGACCATCACGGTTATCCCAGGGCTGAAGACA<br>ATTGTGGGAGCCCTGATCCAGTCTGTGAAAAAGCTGTCGGAT<br>GTGATGATCCTCACTGTCTTCTGCCTGAGTGTCTTTGCCCTG<br>GTGGGGCTGCAGCTTTTCATGGGAAACCTGCGTCAGAAGTGC<br>GTGCGTTGGCCCCCGCCCATGAATGACACCAACACCACGTGG<br>TATGGCAATGACACTTGGTACAGCAATGACACTTGGTACGGC<br>AATGACACTTGGTACATCAATGACACTTGGAACAGCCAGGAG<br>AGCTGGGCCGGCAACTCTACCTTTGACTGGGAGGCCTACATC<br>AATGACGAAGGGAACTTCTATTTCTTGGAGGGCTCCAATGAT

		GCTCTGCTCTGTGGGAATAGCAGTGATGCTGGGCACTGCCCT<br>GAGGGCTACGAATGCATAAAGGCTGGGCGGAACCCCAACTAT<br>GGCTACACCAGCTATGACACCTTCAGCTGGGCTTTCCTGGCT<br>CTCTTCCGGCTCATGACGCAGGACTACTGGGAGAACCTTTTC<br>CAGCTGACCCTACGAGCTGCTGGCAAGACCTACATGATCTTC<br>TTCGTGGTCATCATCTTCCTGGGCTCCTTCTACCTCATCAAT<br>CTGATCCTGGCCGTGGTGGCCATGGCGTACGCTGAGCAGAAT<br>GAGGCTACCCTGGCCGAAGACCAGGAGAAAGAGGAGGAGTTC<br>CAACAGATGCTTGAGAAATACAAAAAACATCAGGAGGAACTG<br>GAAAAGGCTAAGGCTGCCCAGGCTCTGGAAAGTGGAGAGGAG<br>GCAGATGGGGACCCAACCCACAACAAAGACTGCAATGGGAGC<br>CTGGATGCATCCGGGGAGAAGGGGCCCCCAAGGCCAAGCTGC<br>AGCGCAGACAGTGCCATCTCAGATGCTATGGAGGAGCTGGAA<br>GAGGCCCATCAGAAGTGCCCACCGTGGTGGTACAAGTGTGCA<br>CACAAAGTCCTCATCTGGAACTGCTGTGCCCCGTGGGTGAAG<br>TTCAAACATATAATCTACCTGATCGTCATGGACCCCTTTGTG<br>GACCTGGGTATCACCATCTGCATTGTGCTCAACACCCTCTTC<br>ATGGCCATGGAGCACTACCCCATGACCGAGCACTTTGACAAC<br>GTGCTCTCCGTGGGCAACTTGGTCTTCACAGGCATCTTCACT<br>GCGGAGATGGTGTTGAAGCTGATTGCCATGGACCCCTACGAG<br>TATTTCCAACAGGGCTGGAACATCTTTGACAGTTTCATCGTC<br>ACCCTCAGCCTGGTGGAGCTGGGCCTGGCCAACGTACAGGGG<br>CTGTCAGTGCTCCGTTCCTTCCGCCTGCTGCGTGTCTTCAAG<br>CTGGCCAAGTCATGGCCAACACTCAACATGCTCATCAAAATC<br>ATTGGCAACTCAGTGGGCGCGCTGGGCAACCTGACCCTGGTG<br>CTGGCCATCATCGTCTTCATCTTCGCCGTGGTGGGCATGCAG<br>CTGTTCGGCAAGAGCTATAAGGAGTGTGTGTGCAAGATCGCC<br>TCAGACTGCAACCTGCCTCGCTGGCACATGAACGACTTCTTC<br>CACTCCTTCCTCATCGTCTTCCGCATCCTCTGCGGGGAATGG<br>ATCGAGACCATGTGGGACTGCATGGAGGTGGCCGGCCAGGCC<br>ATGTGCCTCACCGTCTTCCTCATGGTCATGGTCATTGGCAAC<br>CTGGTGGTCCTGAATCTGTTCCTGGCTCTCCTGCTGAGTTCC<br>TTCAGTGCTGACAGCCTGGCGGCCTCGGATGAGGATGGCGAG<br>ATGAACAACCTACAGATTGCCATCGGGCGTATCAAGTGGGGC<br>ATCGGTTTTGCCAAAACCTTCCTCCTGGGGCTGTTGCGTGGC<br>AAGATCCTGAGCCCCAAGGAAATAATACTCAGCCTCGGTGAG<br>CCCGGGGGTGCCGGGGAAAATGCCGAGGAGAGTACTCCCGAG<br>GATGAGAAGAAGGAGCCGCCACCGGAAGATAAGGAGCTGAAA<br>GACAATCATATCCTGAACCACGTGGGCCTGACCGATGGCCCC<br>CGCTCCAGCATCGAGCTGGACCACCTTAACTTCATCAACAAC<br>CCCTACCTCACCATCCAGGTGCCCATTGCCTCCGAGGAGTCT<br>GACCTGGAGATGCCCACAGAGGAGGAGACAGACGCCTTCTCG<br>GAGCCTGAGGATATCAAGAAGCCCCTACAGCCCCTCTACGAC<br>GGGAACTCCTCCGTCTGCAGCACAGCTGACTACAAGCCCCCT

		GAAGAGGACCCCGAGGAGCAGGCTGAGGAGAACCCCGAGGGG<br>GAGCAGCCTGAGGAATGCTTCACGGAAGCCTGTGTGAAGCGC<br>TGCCCCTGC
Insert-10	three 1 kb	ATGGCAAACTTCCTATTACCTCGGGGCACCAGCAGCTTCCGC<br>AGGTTCACACGGGAGTCCCTGGCAGCCATCGAGAAGCGCATG<br>GCGGAGAAGCAAGCCCGCGGCTCAACCACCTTGCAGGAGAGC<br>CGAGAGGGGCTGCCCGAGGAGGAGGCTCCCCGGCCCCAGCTG<br>GACCTGCAGGCCTCCAAAAAGCTGCCAGATCTCTATGGCAAT<br>CCACCCCAAGAGCTCATCGGAGAGCCCCTGGAGGACCTGGAC<br>CCCTTCTATAGCACCCAAAAGACTTTCATCGTACTGAATAAA<br>GGCAAGACCATCTTCCGGTTCAGTGCCACCAACGCCTTGTAT<br>GTCCTCAGTCCCTTCCACCCCATCCGGAGAGCGGCTGTGAAG<br>ATTCTGGTTCACTCGCTCTTCAACATGCTCATCATGTGCACC<br>ATCCTCACCAACTGCGTGTTCATGGCCCAGCACGACCCTCCA<br>CCCTGGACCAAGTATGTCGAGTACACCTTCACCGCCATTTAC<br>ACCTTTGAGTCTCTGGTCAAGATTCTGGCTCGAGGCTTCTGC<br>CTGCACGCGTTCACTTTCCTTCGGGACCCATGGAACTGGCTG<br>GACTTTAGTGTGATTATCATGGCATACACAACTGAATTTGTG<br>GACCTGGGCAATGTCTCAGCCTTACGCACCTTCCGAGTCCTC<br>CGGGCCCTGAAAACTATATCAGTCATTTCAGGGCTGAAGACC<br>ATCGTGGGGGCCCTGATCCAGTCTGTGAAGAAGCTGGCTGAT<br>GTGATGGTCCTCACAGTCTTCTGCCTCAGCGTCTTTGCCCTC<br>ATCGGCCTGCAGCTCTTCATGGGCAACCTAAGGCACAAGTGC<br>GTGCGCAACTTCACAGCGCTCAACGGCACCAACGGCTCCGTG<br>GAGGCCGACGGCTTGGTCTGGGAATCCCTGGACCTTTACCTC<br>AGTGATCCAGAAAATTACCTGCTCAAGAACGGCACCTCTGAT<br>GTGTTACTGTGTGGGAACAGCTCTGACGCTGGGA<br>ATGGCCAGCTCATCTCTGCCCAACCTGGTCCCCCCGGGTCCC<br>CACTGCCTGCGCCCCTTCACCCCAGAGTCCCTGGCAGCCATA<br>GAGCAGCGGGCGGTGGAGGAGGAGGCCCGGCTGCAGCGGAAC<br>AAGCAGATGGAGATTGAGGAGCCTGAGCGGAAGCCACGCAGT<br>GACCTGGAAGCTGGCAAGAACCTCCCACTCATCTATGGGGAC<br>CCCCCACCCGAAGTCATTGGCATCCCCCTGGAGGACCTGGAT<br>CCTTACTACAGTGACAAGAAGACCTTCATTGTGCTCAACAAA<br>GGAAAGGCCATCTTCCGATTCTCTGCCACGCCTGCCCTCTAC<br>CTGCTGAGCCCCTTCAGCATCGTCAGGAGGGTGGCTATCAAG<br>GTGCTCATTCACGCGCTGTTCAGCATGTTTATCATGATCACC<br>ATCCTGACCAACTGTGTGTTCATGACCATGAGCAATCCGCCT<br>TCTTGGTCCAAACACGTGGAGTACACCTTCACGGGGATCTAT<br>ACCTTTGAGTCCCTCATTAAGATGCTGGCCCGAGGCTTTTGC<br>ATTGATGACTTCACATTCCTCCGAGACCCCTGGAACTGGCTG<br>GACTTCAGTGTCATCACAATGGCGTATGTGACAGAGTTTGTG<br>GACTTGGGCAACATCTCAGCCCTGAGGACCTTCCGTGTGCTG<br>CGGGCCCTGAAGACCATCACGGTTATCCCAGGGCTGAAGACA

		ATTGTGGGAGCCCTGATCCAGTCTGTGAAAAAGCTGTCGGAT<br>GTGATGATCCTCACTGTCTTCTGCCTGAGTGTCTTTGCCCTG<br>GTGGGGCTGCAGCTTTTCATGGGAAACCTGCGTCAGAAGTGC<br>GTGCGTTGGCCCCCGCCCATGAATGACACCAACACCACGTGG<br>TATGGCAATGACACTTGGTACAGCAATGACACTTGGTACGGC<br>AATGACACTTGGTACATCAATGACACTTGGAACAGCCAGGAG<br>AGCTGGGCCGGCAACTCTACCTTTGACTGGGAGG<br>GCGTCCTCACCAGCGCACTGGAAGAGTTAGAGGAGTCTCGCC<br>ATAAGTGTCCACCATGCTGGAACCGTCTCGCCCAGCGCTACC<br>TGATCTGGGAGTGCTGCCCGCTGTGGATGTCCATCAAGCAGG<br>GAGTGAAGTTGGTGGTCATGGACCCGTTTACTGACCTCACCA<br>TCACTATGTGCATCGTACTCAACACACTCTTCATGGCGCTGG<br>AGCACTACAACATGACAAGTGAATTCGAGGAGATGCTGCAGG<br>TCGGAAACCTGGTCTTCACAGGGATTTTCACAGCAGAGATGA<br>CCTTCAAGATCATTGCCCTCGACCCCTACTACTACTTCCAAC<br>AGGGCTGGAACATCTTCGACAGCATCATCGTCATCCTTAGCC<br>TCATGGAGCTGGGCCTGTCCCGCATGAGCAACTTGTCGGTGC<br>TGCGCTCCTTCCGCCTGCTGCGGGTCTTCAAGCTGGCCAAAT<br>CATGGCCCACCCTGAACACACTCATCAAGATCATCGGGAACT<br>CAGTGGGGGCACTGGGGAACCTGACACTGGTGCTAGCCATCA<br>TCGTGTTCATCTTTGCTGTGGTGGGCATGCAGCTCTTTGGCA<br>AGAACTACTCGGAGCTGAGGGACAGCGACTCAGGCCTGCTGC<br>CTCGCTGGCACATGATGGACTTCTTTCATGCCTTCCTCATCA<br>TCTTCCGCATCCTCTGTGGAGAGTGGATCGAGACCATGTGGG<br>ACTGCATGGAGGTGTCGGGGCAGTCATTATGCCTGCTGGTCT<br>TCTTGCTTGTTATGGTCATTGGCAACCTTGTGGTCCTGAATC<br>TCTTCCTGGCCTTGCTGCTCAGCTCCTTCAGTGCAGACAACC<br>TCACAGCCCCTGATGAGGACAGAGAAGATGAACAACCTCCAA<br>GCTGGCCCTGGCCCGCATCCAGAAGGGGCCTGCGCTTTGTCA<br>AGCGGACCACCTGGGATTTCTGCTGTGGTCTCCTGCGGCAGC<br>GGCCTCAGAAGCCCGCAGCCCTTGCCGC
Insert-11	three 2 kb	ATGGCAAACTTCCTATTACCTCGGGGCACCAGCAGCTTCCGC<br>AGGTTCACACGGGAGTCCCTGGCAGCCATCGAGAAGCGCATG<br>GCGGAGAAGCAAGCCCGCGGCTCAACCACCTTGCAGGAGAGC<br>CGAGAGGGGCTGCCCGAGGAGGAGGCTCCCCGGCCCCAGCTG<br>GACCTGCAGGCCTCCAAAAAGCTGCCAGATCTCTATGGCAAT<br>CCACCCCAAGAGCTCATCGGAGAGCCCCTGGAGGACCTGGAC<br>CCCTTCTATAGCACCCAAAAGACTTTCATCGTACTGAATAAA<br>GGCAAGACCATCTTCCGGTTCAGTGCCACCAACGCCTTGTAT<br>GTCCTCAGTCCCTTCCACCCCATCCGGAGAGCGGCTGTGAAG<br>ATTCTGGTTCACTCGCTCTTCAACATGCTCATCATGTGCACC<br>ATCCTCACCAACTGCGTGTTCATGGCCCAGCACGACCCTCCA<br>CCCTGGACCAAGTATGTCGAGTACACCTTCACCGCCATTTAC<br>ACCTTTGAGTCTCTGGTCAAGATTCTGGCTCGAGGCTTCTGC

		CTGCACGCGTTCACTTTCCTTCGGGACCCATGGAACTGGCTG<br>GACTTTAGTGTGATTATCATGGCATACACAACTGAATTTGTG<br>GACCTGGGCAATGTCTCAGCCTTACGCACCTTCCGAGTCCTC<br>CGGGCCCTGAAAACTATATCAGTCATTTCAGGGCTGAAGACC<br>ATCGTGGGGGCCCTGATCCAGTCTGTGAAGAAGCTGGCTGAT<br>GTGATGGTCCTCACAGTCTTCTGCCTCAGCGTCTTTGCCCTC<br>ATCGGCCTGCAGCTCTTCATGGGCAACCTAAGGCACAAGTGC<br>GTGCGCAACTTCACAGCGCTCAACGGCACCAACGGCTCCGTG<br>GAGGCCGACGGCTTGGTCTGGGAATCCCTGGACCTTTACCTC<br>AGTGATCCAGAAAATTACCTGCTCAAGAACGGCACCTCTGAT<br>GTGTTACTGTGTGGGAACAGCTCTGACGCTGGGACATGTCCG<br>GAGGGCTACCGGTGCCTAAAGGCAGGCGAGAACCCCGACCAC<br>GGCTACACCAGCTTCGATTCCTTTGCCTGGGCCTTTCTTGCA<br>CTCTTCCGCCTGATGACGCAGGACTGCTGGGAGCGCCTCTAT<br>CAGCAGACCCTCAGGTCCGCAGGGAAGATCTACATGATCTTC<br>TTCATGCTTGTCATCTTCCTGGGGTCCTTCTACCTGGTGAAC<br>CTGATCCTGGCCGTGGTCGCAATGGCCTATGAGGAGCAAAAC<br>CAAGCCACCATCGCTGAGACCGAGGAGAAGGAAAAGCGCTTC<br>CAGGAGGCCATGGAAATGCTCAAGAAAGAACACGAGGCCCTC<br>ACCATCAGGGGTGTGGATACCGTGTCCCGTAGCTCCTTGGAG<br>ATGTCCCCTTTGGCCCCAGTAAACAGCCATGAGAGAAGAAGC<br>AAGAGGAGAAAACGGATGTCTTCAGGAACTGAGGAGTGTGGG<br>GAGGACAGGCTCCCCAAGTCTGACTCAGAAGATGGTCCCAGA<br>GCAATGAATCATCTCAGCCTCACCCGTGGCCTCAGCAGGACT<br>TCTATGAAGCCACGTTCCAGCCGCGGGAGCATTTTCACCTTT<br>CGCAGGCGAGACCTGGGTTCTGAAGCAGATTTTGCAGATGAT<br>GAAAACAGCACAGCGGGGGAGAGCGAGAGCCACCACGCATC<br>ACTGCTGGTGCCCTGGCCCCTGCGCCGGACCAGTGCCCAGGG<br>ACAGCCCAGTCCCGGAACCTCGGCTCCTGGCCACGCCCTCCA<br>TGGCAAAAAGAACAGCACTGTGGACTGCAATGGGGTGGTCTC<br>ATTACTGGGGGCAGGCGACCCAGAGGCCACATCCCCAGGAAG<br>CCACCTCCTCCGCCCTGTGATGCTAGAGCACCCGCCAGACAC<br>GACCACGCCATCGGAGGAGCCAGGCGGGCCCCAGATGCTGAC<br>CTCCCAGGCTCCGTGTGTAGATGGCTTCGAGGAGCCAGGAGC<br>ACGGCAGCGGGCCCTCAGCGCAGTCA<br>ATGGCCAGCTCATCTCTGCCCAACCTGGTCCCCCCGGGTCCC<br>CACTGCCTGCGCCCCTTCACCCCAGAGTCCCTGGCAGCCATA<br>GAGCAGCGGGCGGTGGAGGAGGAGGCCCGGCTGCAGCGGAAC<br>AAGCAGATGGAGATTGAGGAGCCTGAGCGGAAGCCACGCAGT<br>GACCTGGAAGCTGGCAAGAACCTCCCACTCATCTATGGGGAC<br>CCCCCACCCGAAGTCATTGGCATCCCCCTGGAGGACCTGGAT<br>CCTTACTACAGTGACAAGAAGACCTTCATTGTGCTCAACAAA<br>GGAAAGGCCATCTTCCGATTCTCTGCCACGCCTGCCCTCTAC<br>CTGCTGAGCCCCTTCAGCATCGTCAGGAGGGTGGCTATCAAG

		GTGCTCATTCACGCGCTGTTCAGCATGTTTATCATGATCACC<br>ATCCTGACCAACTGTGTGTTCATGACCATGAGCAATCCGCCT<br>TCTTGGTCCAAACACGTGGAGTACACCTTCACGGGGATCTAT<br>ACCTTTGAGTCCCTCATTAAGATGCTGGCCCGAGGCTTTTGC<br>ATTGATGACTTCACATTCCTCCGAGACCCCTGGAACTGGCTG<br>GACTTCAGTGTCATCACAATGGCGTATGTGACAGAGTTTGTG<br>GACTTGGGCAACATCTCAGCCCTGAGGACCTTCCGTGTGCTG<br>CGGGCCCTGAAGACCATCACGGTTATCCCAGGGCTGAAGACA<br>ATTGTGGGAGCCCTGATCCAGTCTGTGAAAAAGCTGTCGGAT<br>GTGATGATCCTCACTGTCTTCTGCCTGAGTGTCTTTGCCCTG<br>GTGGGGCTGCAGCTTTTCATGGGAAACCTGCGTCAGAAGTGC<br>GTGCGTTGGCCCCCGCCCATGAATGACACCAACACCACGTGG<br>TATGGCAATGACACTTGGTACAGCAATGACACTTGGTACGGC<br>AATGACACTTGGTACATCAATGACACTTGGAACAGCCAGGAG<br>AGCTGGGCCGGCAACTCTACCTTTGACTGGGAGGCCTACATC<br>AATGACGAAGGGAACTTCTATTTCTTGGAGGGCTCCAATGAT<br>GCTCTGCTCTGTGGGAATAGCAGTGATGCTGGGCACTGCCCT<br>GAGGGCTACGAATGCATAAAGGCTGGGCGGAACCCCAACTAT<br>GGCTACACCAGCTATGACACCTTCAGCTGGGCTTTCCTGGCT<br>CTCTTCCGGCTCATGACGCAGGACTACTGGGAGAACCTTTTC<br>CAGCTGACCCTACGAGCTGCTGGCAAGACCTACATGATCTTC<br>TTCGTGGTCATCATCTTCCTGGGCTCCTTCTACCTCATCAAT<br>CTGATCCTGGCCGTGGTGGCCATGGCGTACGCTGAGCAGAAT<br>GAGGCTACCCTGGCCGAAGACCAGGAGAAAGAGGAGGAGTTC<br>CAACAGATGCTTGAGAAATACAAAAAACATCAGGAGGAACTG<br>GAAAAGGCTAAGGCTGCCCAGGCTCTGGAAAGTGGAGAGGAG<br>GCAGATGGGGACCCAACCCACAACAAAGACTGCAATGGGAGC<br>CTGGATGCATCCGGGGAGAAGGGGCCCCCAAGGCCAAGCTGC<br>AGCGCAGACAGTGCCATCTCAGATGCTATGGAGGAGCTGGAA<br>GAGGCCCATCAGAAGTGCCCACCGTGGTGGTACAAGTGTGCA<br>CACAAAGTCCTCATCTGGAACTGCTGTGCCCCGTGGGTGAAG<br>TTCAAACATATAATCTACCTGATCGTCATGGACCCCTTTGTG<br>GACCTGGGTATCACCATCTGCATTGTGCTCAACACCCTCTTC<br>ATGGCCATGGAGCACTACCCCATGACCGAGCACTTTGACAAC<br>GTGCTCTCCGTGGGCAACTTGGTCTTCACAGGCATCTTCACT<br>GCGGAGATGGTGTTGAAGCTGATTGCCATGGACCCCTACGAG<br>TATTTCCAACAGGGCTGGAACATCTTTGACAGTTTCATCGTC<br>ACCCTCAGCCTGGTGGAGCTGGGCCTGGCCAACGTACAGGGG<br>CTGTCAGTGCTCCGTTCCTTCCGC<br>GCGTCCTCACCAGCGCACTGGAAGAGTTAGAGGAGTCTCGCC<br>ATAAGTGTCCACCATGCTGGAACCGTCTCGCCCAGCGCTACC<br>TGATCTGGGAGTGCTGCCCGCTGTGGATGTCCATCAAGCAGG<br>GAGTGAAGTTGGTGGTCATGGACCCGTTTACTGACCTCACCA<br>TCACTATGTGCATCGTACTCAACACACTCTTCATGGCGCTGG

		AGCACTACAACATGACAAGTGAATTCGAGGAGATGCTGCAGG<br>TCGGAAACCTGGTCTTCACAGGGATTTTCACAGCAGAGATGA<br>CCTTCAAGATCATTGCCCTCGACCCCTACTACTACTTCCAAC<br>AGGGCTGGAACATCTTCGACAGCATCATCGTCATCCTTAGCC<br>TCATGGAGCTGGGCCTGTCCCGCATGAGCAACTTGTCGGTGC<br>TGCGCTCCTTCCGCCTGCTGCGGGTCTTCAAGCTGGCCAAAT<br>CATGGCCCACCCTGAACACACTCATCAAGATCATCGGGAACT<br>CAGTGGGGGCACTGGGGAACCTGACACTGGTGCTAGCCATCA<br>TCGTGTTCATCTTTGCTGTGGTGGGCATGCAGCTCTTTGGCA<br>AGAACTACTCGGAGCTGAGGGACAGCGACTCAGGCCTGCTGC<br>CTCGCTGGCACATGATGGACTTCTTTCATGCCTTCCTCATCA<br>TCTTCCGCATCCTCTGTGGAGAGTGGATCGAGACCATGTGGG<br>ACTGCATGGAGGTGTCGGGGCAGTCATTATGCCTGCTGGTCT<br>TCTTGCTTGTTATGGTCATTGGCAACCTTGTGGTCCTGAATC<br>TCTTCCTGGCCTTGCTGCTCAGCTCCTTCAGTGCAGACAACC<br>TCACAGCCCCTGATGAGGACAGAGAAGATGAACAACCTCCAA<br>GCTGGCCCTGGCCCGCATCCAGAAGGGGCCTGCGCTTTGTCA<br>AGCGGACCACCTGGGATTTCTGCTGTGGTCTCCTGCGGCAGC<br>GGCCTCAGAAGCCCGCAGCCCTTGCCGCCCAGGGCCAGCTGC<br>CCAGCTGCATTGCCACCCCCTACTCCCCGCCACCCCCAGAGA<br>CGGAGAAGGTGCCTCCCACCCGCAAGGAAACACGGTTTGAGG<br>AAGGCGAGCAACCAGGCCAGGGCACCCCCGGGGATCCAGAGC<br>CCGTGTGTGTGCCCATCGCTGTGGCCGAGTCAGACACAGATG<br>ACCAAGAAGAAGATGAGGAGAACAGCCTGGGCACGGAGGAGG<br>AGTCCAGCAAGCAGGAATCCCAGCCTGTGTCCGGTGGCCCAG<br>AGGCCCCTCCGGATTCCAGGACCTGGAGCCAGGTGTCAGCGA<br>CTGCCTCCTCTGAGGCCGAGGCCAGTGCATCTCAGGCCGACT<br>GGCGGCAGCAGTGGAAAGCGGAACCCCAGGCCCCAGGGTGCG<br>GTGAGACCCCAGAGGACAGTTGCTCCGAGGGCAGCACAGCAG<br>ACATGACCAACACCGCTGAGCTCCTGGAGCAGATCCCTGACC<br>TCGGCCAGGATGTCAAGGACCCAGAGGACTGCTTCACTGAAG<br>GCTGTGTCCGGCGCTGTCCCTGCTGTGCGGTGGACACCACAC<br>AGGCCCCAGGGAAGGTCTGGTGGCGGTTGCGCAAGACCTGCT<br>ACCACATCGTGGAGCACAGCTGGTTCGAGACATTCATCATCT<br>TCATGATCCTACTCAGCAGTGGAGCGCTGGCCTTCGAGGACA<br>TCTACCTAGAGGAGCGGAAGACCATCAAGGTTCTGCTTGAGT<br>ATGCCGACAAGATGTTCACATATGTCTTCGTGCTGGAGATGC<br>TGCTCAAGTGGGTGGCCTACGGCTTCAAGAAGTACTTCACCA<br>ATGCCTGGTGCTGGCTCGACTTCCTCATCGTAGACGTCTCTC<br>TGGTCAGCCTGGTGGCCAACACCCTGGGCTTTGCCGAGATGG<br>GCCCCATCAAGTCACTGCGGACGCTGCGTGCACTCCGTCCTC<br>TGAGAGCTCTGTCACGATTTGAGGGCATGAGGGTGGTGGTCA<br>ATGCCCTGGTGGGCGCCA
Insert-	four 1 kb	ATGGCAAACTTCCTATTACCTCGGGGCACCAGCAGCTTCCGC

12		AGGTTCACACGGGAGTCCCTGGCAGCCATCGAGAAGCGCATG<br>GCGGAGAAGCAAGCCCGCGGCTCAACCACCTTGCAGGAGAGC<br>CGAGAGGGGCTGCCCGAGGAGGAGGCTCCCCGGCCCCAGCTG<br>GACCTGCAGGCCTCCAAAAAGCTGCCAGATCTCTATGGCAAT<br>CCACCCCAAGAGCTCATCGGAGAGCCCCTGGAGGACCTGGAC<br>CCCTTCTATAGCACCCAAAAGACTTTCATCGTACTGAATAAA<br>GGCAAGACCATCTTCCGGTTCAGTGCCACCAACGCCTTGTAT<br>GTCCTCAGTCCCTTCCACCCCATCCGGAGAGCGGCTGTGAAG<br>ATTCTGGTTCACTCGCTCTTCAACATGCTCATCATGTGCACC<br>ATCCTCACCAACTGCGTGTTCATGGCCCAGCACGACCCTCCA<br>CCCTGGACCAAGTATGTCGAGTACACCTTCACCGCCATTTAC<br>ACCTTTGAGTCTCTGGTCAAGATTCTGGCTCGAGGCTTCTGC<br>CTGCACGCGTTCACTTTCCTTCGGGACCCATGGAACTGGCTG<br>GACTTTAGTGTGATTATCATGGCATACACAACTGAATTTGTG<br>GACCTGGGCAATGTCTCAGCCTTACGCACCTTCCGAGTCCTC<br>CGGGCCCTGAAAACTATATCAGTCATTTCAGGGCTGAAGACC<br>ATCGTGGGGGCCCTGATCCAGTCTGTGAAGAAGCTGGCTGAT<br>GTGATGGTCCTCACAGTCTTCTGCCTCAGCGTCTTTGCCCTC<br>ATCGGCCTGCAGCTCTTCATGGGCAACCTAAGGCACAAGTGC<br>GTGCGCAACTTCACAGCGCTCAACGGCACCAACGGCTCCGTG<br>GAGGCCGACGGCTTGGTCTGGGAATCCCTGGACCTTTACCTC<br>AGTGATCCAGAAAATTACCTGCTCAAGAACGGCACCTCTGAT<br>GTGTTACTGTGTGGGAACAGCTCTGACGCTGGGA<br>ATGGCCAGCTCATCTCTGCCCAACCTGGTCCCCCCGGGTCCC<br>CACTGCCTGCGCCCCTTCACCCCAGAGTCCCTGGCAGCCATA<br>GAGCAGCGGGCGGTGGAGGAGGAGGCCCGGCTGCAGCGGAAC<br>AAGCAGATGGAGATTGAGGAGCCTGAGCGGAAGCCACGCAGT<br>GACCTGGAAGCTGGCAAGAACCTCCCACTCATCTATGGGGAC<br>CCCCCACCCGAAGTCATTGGCATCCCCCTGGAGGACCTGGAT<br>CCTTACTACAGTGACAAGAAGACCTTCATTGTGCTCAACAAA<br>GGAAAGGCCATCTTCCGATTCTCTGCCACGCCTGCCCTCTAC<br>CTGCTGAGCCCCTTCAGCATCGTCAGGAGGGTGGCTATCAAG<br>GTGCTCATTCACGCGCTGTTCAGCATGTTTATCATGATCACC<br>ATCCTGACCAACTGTGTGTTCATGACCATGAGCAATCCGCCT<br>TCTTGGTCCAAACACGTGGAGTACACCTTCACGGGGATCTAT<br>ACCTTTGAGTCCCTCATTAAGATGCTGGCCCGAGGCTTTTGC<br>ATTGATGACTTCACATTCCTCCGAGACCCCTGGAACTGGCTG<br>GACTTCAGTGTCATCACAATGGCGTATGTGACAGAGTTTGTG<br>GACTTGGGCAACATCTCAGCCCTGAGGACCTTCCGTGTGCTG<br>CGGGCCCTGAAGACCATCACGGTTATCCCAGGGCTGAAGACA<br>ATTGTGGGAGCCCTGATCCAGTCTGTGAAAAAGCTGTCGGAT<br>GTGATGATCCTCACTGTCTTCTGCCTGAGTGTCTTTGCCCTG<br>GTGGGGCTGCAGCTTTTCATGGGAAACCTGCGTCAGAAGTGC<br>GTGCGTTGGCCCCCGCCCATGAATGACACCAACACCACGTGG

		TATGGCAATGACACTTGGTACAGCAATGACACTTGGTACGGC<br>AATGACACTTGGTACATCAATGACACTTGGAACAGCCAGGAG<br>AGCTGGGCCGGCAACTCTACCTTTGACTGGGAGG<br>GCGTCCTCACCAGCGCACTGGAAGAGTTAGAGGAGTCTCGCC<br>ATAAGTGTCCACCATGCTGGAACCGTCTCGCCCAGCGCTACC<br>TGATCTGGGAGTGCTGCCCGCTGTGGATGTCCATCAAGCAGG<br>GAGTGAAGTTGGTGGTCATGGACCCGTTTACTGACCTCACCA<br>TCACTATGTGCATCGTACTCAACACACTCTTCATGGCGCTGG<br>AGCACTACAACATGACAAGTGAATTCGAGGAGATGCTGCAGG<br>TCGGAAACCTGGTCTTCACAGGGATTTTCACAGCAGAGATGA<br>CCTTCAAGATCATTGCCCTCGACCCCTACTACTACTTCCAAC<br>AGGGCTGGAACATCTTCGACAGCATCATCGTCATCCTTAGCC<br>TCATGGAGCTGGGCCTGTCCCGCATGAGCAACTTGTCGGTGC<br>TGCGCTCCTTCCGCCTGCTGCGGGTCTTCAAGCTGGCCAAAT<br>CATGGCCCACCCTGAACACACTCATCAAGATCATCGGGAACT<br>CAGTGGGGGCACTGGGGAACCTGACACTGGTGCTAGCCATCA<br>TCGTGTTCATCTTTGCTGTGGTGGGCATGCAGCTCTTTGGCA<br>AGAACTACTCGGAGCTGAGGGACAGCGACTCAGGCCTGCTGC<br>CTCGCTGGCACATGATGGACTTCTTTCATGCCTTCCTCATCA<br>TCTTCCGCATCCTCTGTGGAGAGTGGATCGAGACCATGTGGG<br>ACTGCATGGAGGTGTCGGGGCAGTCATTATGCCTGCTGGTCT<br>TCTTGCTTGTTATGGTCATTGGCAACCTTGTGGTCCTGAATC<br>TCTTCCTGGCCTTGCTGCTCAGCTCCTTCAGTGCAGACAACC<br>TCACAGCCCCTGATGAGGACAGAGAAGATGAACAACCTCCAA<br>GCTGGCCCTGGCCCGCATCCAGAAGGGGCCTGCGCTTTGTCA<br>AGCGGACCACCTGGGATTTCTGCTGTGGTCTCCTGCGGCAGC<br>GGCCTCAGAAGCCCGCAGCCCTTGCCGC<br>CCTACATCAATGACGAAGGGAACTTCTATTTCTTGGAGGGCT<br>CCAATGATGCTCTGCTCTGTGGGAATAGCAGTGATGCTGGGC<br>ACTGCCCTGAGGGCTACGAATGCATAAAGGCTGGGCGGAACC<br>CCAACTATGGCTACACCAGCTATGACACCTTCAGCTGGGCTT<br>TCCTGGCTCTCTTCCGGCTCATGACGCAGGACTACTGGGAGA<br>ACCTTTTCCAGCTGACCCTACGAGCTGCTGGCAAGACCTACA<br>TGATCTTCTTCGTGGTCATCATCTTCCTGGGCTCCTTCTACC<br>TCATCAATCTGATCCTGGCCGTGGTGGCCATGGCGTACGCTG<br>AGCAGAATGAGGCTACCCTGGCCGAAGACCAGGAGAAAGAGG<br>AGGAGTTCCAACAGATGCTTGAGAAATACAAAAAACATCAGG<br>AGGAACTGGAAAAGGCTAAGGCTGCCCAGGCTCTGGAAAGTG<br>GAGAGGAGGCAGATGGGGACCCAACCCACAACAAAGACTGCA<br>ATGGGAGCCTGGATGCATCCGGGGAGAAGGGGCCCCCAAGGC<br>CAAGCTGCAGCGCAGACAGTGCCATCTCAGATGCTATGGAGG<br>AGCTGGAAGAGGCCCATCAGAAGTGCCCACCGTGGTGGTACA<br>AGTGTGCACACAAAGTCCTCATCTGGAACTGCTGTGCCCCGT<br>GGGTGAAGTTCAAACATATAATCTACCTGATCGTCATGGACC

		CCTTTGTGGACCTGGGTATCACCATCTGCATTGTGCTCAACA<br>CCCTCTTCATGGCCATGGAGCACTACCCCATGACCGAGCACT<br>TTGACAACGTGCTCTCCGTGGGCAACTTGGTCTTCACAGGCA<br>TCTTCACTGCGGAGATGGTGTTGAAGCTGATTGCCATGGACC<br>CCTACGAGTATTTCCAACAGGGCTGGAACATCTTTGACAGTT<br>TCATCGTCACCCTCAGCCTGGTGGAGCTGGGCCTGGCCAACG<br>TACAGGGGCTG<u>TCAGTGCTCCGTTCCTTCCGC</u>
Insertion site		TAATACGACTCACTATAGGGCGAGCGCCGCCATGGAGTACCC<br>ATACGACGTACCAGATTACG<u>CTCATATGGCCATGGAGGCCAG</u><br><u>TGAA</u><br>Insertion site<br><u>TTCCACCCGGGTGGGCATCGATAC</u>GGATCCATCGAGCTCGA<br>GCTGCAGATGAATCGTAGATACTGAAAAACCCCGCAAGTTCA<br>CTTCAACTGTGCATCGTGCACCAT

The sequences from hNa$_V$1.5 and rNa$_V$1.4 are shaded in yellow and azure, respectively. The inserts were constructed into the site, between both green and pink shading sequences, of the plasmid pGADT$_7$. The homologous regions of primers to inserts and vector are undelined.

Supplementary Table S2. Primer sequences.

primer name	sequence
F1-0	ATGGCAAACTTCCTATTACCTCGG
F1-5	GTGAAATGGCAAACTTCCTATTACCTCGG
F1-10	GGCCAGTGAAATGGCAAACTTCCTATTACCTCGG
F1-15	ATGGAGGCCAGTGAAATGGCAAACTTCCTATTACCTCGG
F1-20	TGGCCATGGAGGCCAGTGAAATGGCAAACTTCCTATTACCTCGG
F1-25	TCATATGGCCATGGAGGCCAGTGAAATGGCAAACTTCCTATTACCTCGG
F1-30	TACGCTCATATGGCCATGGAGGCCAGTGAAATGGCAAACTTCCTATTACCTCGG
F1-35	CAGATTACGCTCATATGGCCATGGAGGCCAGTGAAATGGCAAACTTCCTATTACCTCGG
1kb-R1	CGATGCCCACCCGGGTGGAATCCCAGCGTCAGAGCTGTTCC
2kb-R1	CGATGCCCACCCGGGTGGAATGACTGCGCTGAGGGCCCGC
3kb-R1	CGATGCCCACCCGGGTGGAAGCGGCAAGGGCTGCGGGCT
4kb-R1	CGATGCCCACCCGGGTGGAATGGCGCCCACCAGGGCATTG
5kb-R1	CGATGCCCACCCGGGTGGAACAGCAGCCCGATGTTGAAGAGG
6kb-R1	CGATGCCCACCCGGGTGGAAGCTGTAGTCAGACCCCCGCA
2X1K-F1-15/30	ATGGAGGCCAGTGAA**ATGGCAAACTTCCTATTACCTCGG**<br>/TACGCTCATATGGCCATGGAGGCCAGTGAA**ATGGCAAACTTCCTATTACCTCGG**
2X1K-R1	*GGGCAGAGATGAGCTGGCCAT***TCCCAGCGTCAGAGCTGTTCC**
2X1K-F2	**ATGGCCAGCTCATCTCTGCCC**
2X1K-R2	GTATCGATGCCCACCCGGGTGGAA**CCTCCCAGTCAAAGGTAGAGTTG**
2X2K-F1-15/30	ATGGAGGCCAGTGAA**ATGGCAAACTTCCTATTACCTCGG**<br>/TACGCTCATATGGCCATGGAGGCCAGTGAA**ATGGCAAACTTCCTATTACCTCGG**
2X2K-R1	*GGGCAGAGATGAGCTGGCCAT***TGACTGCGCTGAGGGCCCGC**
2X2K-F2	**ATGGCCAGCTCATCTCTGCCC**
2X2K-R2	GTATCGATGCCCACCCGGGTGGAA**GCGGAAGGAACGGAGCACTGA**
2X3K-F1-15/30	ATGGAGGCCAGTGAA**ATGGCAAACTTCCTATTACCTCGG**<br>/TACGCTCATATGGCCATGGAGGCCAGTGAA**ATGGCAAACTTCCTATTACCTCGG**
2X3K-R1	*GGGCAGAGATGAGCTGGCCAT***GCGGCAAGGGCTGCGGGCT**
2X3K-F2	**ATGGCCAGCTCATCTCTGCCC**
2X3K-R2	GTATCGATGCCCACCCGGGTGGAA**GCAGGGGCAGCGCTTCACAC**

3X1K-F1-15/30	ATGGAGGCCAGTGAA**ATGGCAAACTTCCTATTACCTCGG** /TACGCTCATATGGCCATGGAGGCCAGTGAA**ATGGCAAACTTCCTATTACCTCGG**
3X1K-R1	*GGGCAGAGATGAGCTGGCCAT***TCCCAGCGTCAGAGCTGTTCC**
3X1K-F2	**ATGGCCAGCTCATCTCTGCCC**
3X1K-R2	*CAGTGCGCTGGTGAGGACGC***CCTCCCAGTCAAAGGTAGAGTTG**
3X1K-F3	**GCGTCCTCACCAGCGCACTG**
3X1K-R3	GTATCGATGCCCACCCGGGTGGAA**GCGGCAAGGGCTGCGGGCT**
3X2K-F1-15/30	ATGGAGGCCAGTGAA**ATGGCAAACTTCCTATTACCTCGG** /TACGCTCATATGGCCATGGAGGCCAGTGAA**ATGGCAAACTTCCTATTACCTCGG**
3X2K-R1	*GGGCAGAGATGAGCTGGCCAT***TGACTGCGCTGAGGGCCCGC**
3X2K-F2	**ATGGCCAGCTCATCTCTGCCC**
3X2K-R2	*CAGTGCGCTGGTGAGGACGC***GCGGAAGGAACGGAGCACTGA**
3X2K-F3	**GCGTCCTCACCAGCGCACTG**
3X2K-R3	GTATCGATGCCCACCCGGGTGGAA**TGGCGCCCACCAGGGCATTG**
4X1K-F1-15/30	ATGGAGGCCAGTGAA**ATGGCAAACTTCCTATTACCTCGG** /TACGCTCATATGGCCATGGAGGCCAGTGAA**ATGGCAAACTTCCTATTACCTCGG**
4X1K-R1	*GGGCAGAGATGAGCTGGCCAT***TCCCAGCGTCAGAGCTGTTCC**
4X1K-F2	**ATGGCCAGCTCATCTCTGCCC**
4X1K-R2	*CAGTGCGCTGGTGAGGACGC***CCTCCCAGTCAAAGGTAGAGTTG**
4X1K-F3	**GCGTCCTCACCAGCGCACTG**
4X1K-R3	*GTTCCCTTCGTCATTGATGTAGG***GCGGCAAGGGCTGCGGGCT**
4X1K-F4	**CCTACATCAATGACGAAGGGAAC**
4X1K-R4	GTATCGATGCCCACCCGGGTGGAA**GCGGAAGGAACGGAGCACTGA**
reverse primer R	TTCACTGGCCTCCATGGCCATAT

The underlined, bold and italic sequences are homologous to the vector, target gene and adjacent gene, respectively.
